# Transcriptional adaptation upregulates utrophin in Duchenne muscular dystrophy

**DOI:** 10.1038/s41586-024-08539-x

**Published:** 2025-02-12

**Authors:** Lara Falcucci, Christopher M. Dooley, Douglas Adamoski, Thomas Juan, Justin Martinez, Angelina M. Georgieva, Kamel Mamchaoui, Cansu Cirzi, Didier Y. R. Stainier

**Affiliations:** 1 Department of Developmental Genetics, Max Planck Institute for Heart and Lung Research, Bad Nauheim, Germany; 2 German Centre for Cardiovascular Research (DZHK), Partner Site Rhine-Main, Bad Nauheim, Germany; 3 Excellence Cluster Cardio-Pulmonary Institute, Bad Nauheim, Frankfurt, Giessen, Germany; 4 Department of Cardiac Development and Remodeling, Max Planck Institute for Heart and Lung Research, Bad Nauheim, Germany; 5 Sorbonne Université, Inserm, Institut de Myologie, Centre de Recherche en Myologie, Paris, France; 6 Present Address: Department of Immunology, Genetics and Pathology, Uppsala University, Uppsala, Sweden

**Keywords:** Gene regulation, Transcription, Genetics research, Experimental models of disease, RNA splicing

## Abstract

Duchenne muscular dystrophy (DMD) is a muscle-degenerating disease caused by mutations in the *DMD* gene, which encodes the dystrophin protein^1,2^. Utrophin (*UTRN*), the genetic and functional paralogue of *DMD*, is upregulated in some DMD patients^3–5^. To further investigate this *UTRN* upregulation, we first developed an inducible messenger RNA (mRNA) degradation system for *DMD* by introducing a premature termination codon (PTC) in one of its alternatively spliced exons. Inclusion of the PTC-containing exon triggers *DMD* mutant mRNA decay and *UTRN* upregulation. Notably, blocking nonsense-mediated mRNA decay results in the reversal of *UTRN* upregulation, whereas overexpressing *DMD* does not. Furthermore, overexpressing *DMD*^*PTC*^ minigenes in wild-type cells causes *UTRN* upregulation, as does a wild-type *DMD* minigene containing a self-cleaving ribozyme. To place these findings in a therapeutic context, we used splice-switching antisense oligonucleotides (ASOs) to induce the skipping of out-of-frame exons of *DMD*, aiming to introduce PTCs. We found that these ASOs cause *UTRN* upregulation. In addition, when using an ASO to restore the *DMD* reading frame in myotubes derived from a *DMD*^ΔE52^ patient, an actual DMD treatment, *UTRN* upregulation was reduced. Altogether, these results indicate that an mRNA decay-based mechanism called transcriptional adaptation^6–8^ plays a key role in *UTRN* upregulation in *DMD*^PTC^ patients, and they highlight an unexplored therapeutic application of ASOs, as well as ribozymes, in inducing genetic compensation via transcriptional adaptation.

## Abstract (web summary)

Transcriptional adaptation upregulates *UTRN* in Duchenne muscular dystrophy (DMD) patients, as supported by several lines of evidence, including the use of splice-switching antisense oligonucleotides to induce the skipping of out-of-frame exons of the *DMD* gene.

## Main

Duchenne muscular dystrophy (DMD) is an X-linked recessive neuromuscular disease caused by mutations in the *DMD* gene, which encodes dystrophin—a protein that acts as a mechanical link between the cytoskeleton and the extracellular matrix, safeguarding muscle cells from contraction-induced damage^1,2^. FDA-approved treatments for DMD include exon skipping therapies for specific deletions^9^, stop codon read-through compounds such as ataluren^10^ and Elevidys, a recombinant gene therapy delivering micro-dystrophin^11^. Utrophin, encoded by *UTRN*, the paralogue of *DMD*, has been shown to be upregulated at the sarcolemma of skeletal muscles in patients carrying frameshift or nonsense *DMD* mutations that result in premature termination codons (PTCs)^3,4^, as well as in the *mdx* DMD mouse model^12–14^, which harbours a nonsense mutation in exon 23 of the *Dmd* gene. By contrast, patients with in-frame deletions in *DMD* have been reported not to show utrophin upregulation^15^. Preclinical studies have revealed an inverse correlation between utrophin expression and disease course severity in DMD^5,16^. Furthermore, *mdx*/*Dmd* mutant mice show a milder phenotype than the *Utrn*/*Dmd* double mutant mice^12^. Therefore, utrophin upregulation has been proposed to be a compensatory mechanism to partially counteract the lack of dystrophin, making it a promising therapeutic strategy for treating DMD patients. However, the mechanisms behind *UTRN* upregulation have remained largely elusive and are thought to be due to the loss of dystrophin protein^17^. Here, to investigate the mechanisms underlying *UTRN* upregulation in DMD patients carrying frameshift or nonsense alleles, we developed several genetic tools and show that *DMD* mutant messenger RNA (mRNA) decay plays a pivotal role in *UTRN* upregulation through a newly identified cellular response called transcriptional adaptation (TA). Furthermore, we reveal a new application for splice-switching antisense oligonucleotides (ASOs), as well as for ribozymes, to trigger genetic compensation via TA. During TA, mutant mRNA decay can lead to the increased transcription of so-called adapting genes^6–8,18–25^. This process is independent of the loss of protein function, and it can lead to functional compensation in some cases, potentially explaining why frameshift or nonsense mutations resulting in PTCs in critical genes do not cause obvious phenotypes^6–8,18–22^. TA has been identified thus far in zebrafish^6,8,18^, *Caenorhabditis elegans*^19^ and mouse cells in culture^8^, but as of yet TA has not been reported in human cells.

## Enhancing *DMD* E37 inclusion

**Fig. 1:**
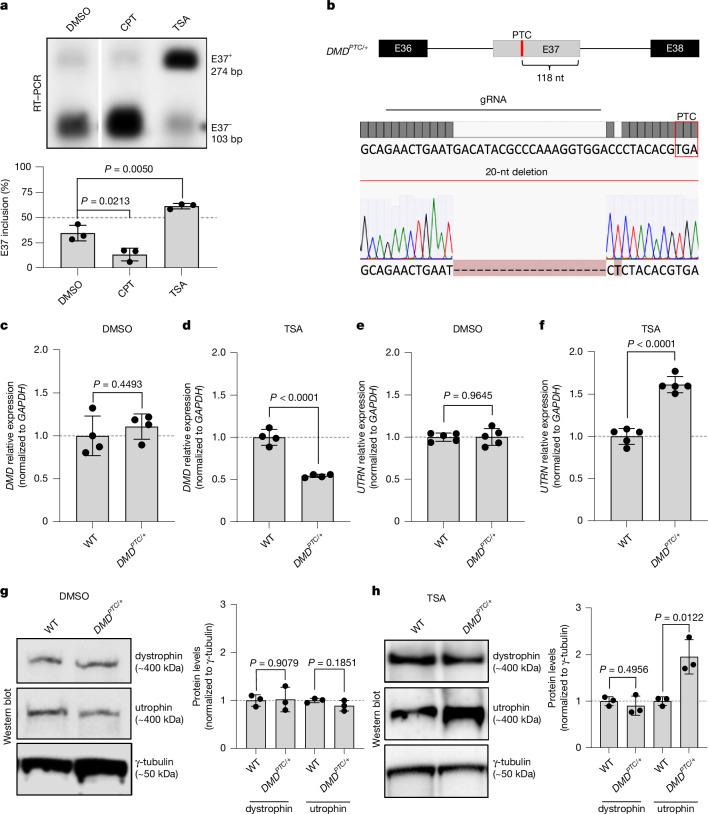
Promoting the inclusion of an alternatively spliced PTC-containing exon trigger*s DMD* mutant mRNA decay and *UTRN* upregulation. **a**, Effects on endogenous *DMD* E37 alternative splicing in WT HEK293T cells after treatment with CPT or TSA; alternative splicing assessed by PCR with reverse transcription (RT–PCR) followed by agarose gel electrophoresis and SYBR Safe staining. Bars display means ± s.d. of the percentage of the intensity of the band containing E37 over the sum of intensity of the bands containing E37 and the skipped isoform (E37^−^) (*n* = 3 biologically independent samples). **b**, Schematic illustration of the *DMD* PTC allele generated with CRISPR–Cas9 in HEK293T cells; red indicates the PTC; gRNA, guide RNA. **c**,**d**, qPCR analysis of *DMD* mRNA levels in WT and *DMD*^*PTC/+*^ cells after treatment with DMSO (**c**) or TSA (**d**) (*n* = 4 biologically independent samples). **e**,**f**, qPCR analysis of *UTRN* mRNA levels in WT and *DMD*^*PTC/+*^ cells after treatment with DMSO (**e**) or TSA (**f**) (*n* = 5 biologically independent samples). **g**,**h**, Western blot analysis and protein quantification showing dystrophin and utrophin levels in WT and *DMD*^*PTC/+*^ cells treated with DMSO (**g**) or TSA (**h**) (*n* = 3 biologically independent samples). Data are normalized to WT and are mean ± s.d.; a two-tailed Student’s *t*-test was used to calculate *P* values. Threshold cycle (Ct) values are included in Supplementary Table 5. Source Data

**Extended Data Fig. 1:**
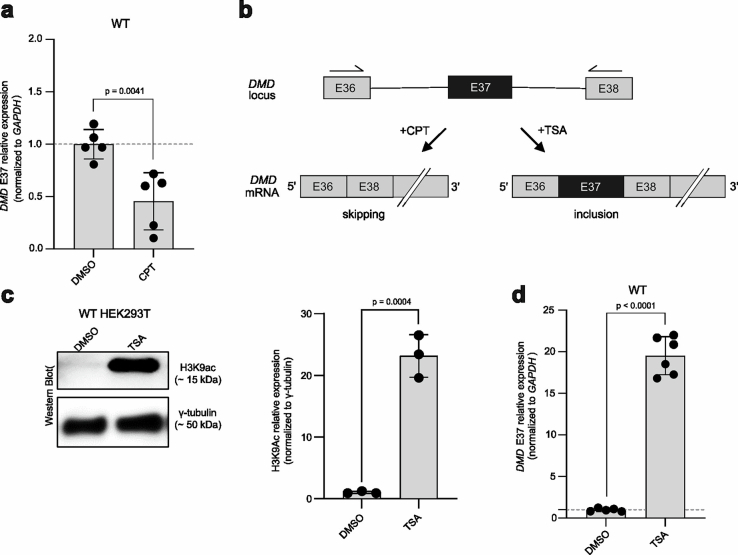
*DMD* E37 is an elongation-sensitive alternatively spliced exon. **a**, qPCR analysis of *DMD* E37 mRNA levels in WT cells after treatment with DMSO or CPT (n = 5 biologically independent samples). **b**, Schematic illustration of the effect of CPT and TSA on *DMD* E37 alternative splicing. Horizontal black half-arrows indicate primers used. **c**, Western blot analysis and quantification of H3K9 acetylation in WT cells after treatment with TSA (n = 3 biologically independent samples). **d**, qPCR analysis of *DMD* E37 mRNA levels in WT cells after treatment with DMSO or TSA (n = 5 biologically independent samples). Data are normalized to DMSO and are mean ± s.d.; a two-tailed Student’s *t*-test was used to calculate p values. Ct values included in Supplementary Table 5. Source Data

To study the role of *DMD* frameshift or nonsense mutations on *UTRN* expression, we first decided to investigate how to modulate the splicing of *DMD* exon 37 (E37). Previous studies have identified frameshift and nonsense mutations in this alternatively spliced exon associated with a milder form of DMD, termed Becker muscular dystrophy^26,27^. Furthermore, bioinformatics analysis has predicted that *DMD* E37 has a weak 3′ splice site and a low exonic splicing enhancer density, thereby leading to frequent exon skipping^28^. To evaluate whether the transcriptional elongation rate has an effect on *DMD* E37 inclusion, we first assessed the effect of camptothecin (CPT), a DNA topoisomerase I inhibitor, previously shown to indirectly inhibit transcriptional elongation^29,30^. We treated wild-type (WT) human embryonic kidney 293T (HEK293T) cells with 3 µM CPT for 6 h, a treatment condition that has been shown not to completely shut down transcription^31,32^, and observed E37 skipping (Fig. 1a and Extended Data Fig. 1a), suggesting that inclusion of this exon follows the kinetic model of cotranscriptional splicing^31,33^. As inhibiting elongation caused E37 skipping, we reasoned that stimulating RNAPII elongation would cause the opposite effect (Extended Data Fig. 1b). Therefore, we treated WT HEK293T cells with 1 µM of the histone deacetylase inhibitor trichostatin A (TSA) for 24 h (Extended Data Fig. 1c), a treatment condition that creates a more relaxed chromatin structure, thereby enhancing elongation^31,33^. We did indeed observe that TSA promotes E37 inclusion (Fig. 1a and Extended Data Fig. 1d).

## Introduction of a PTC in *DMD* E37

**Extended Data Fig. 2:**
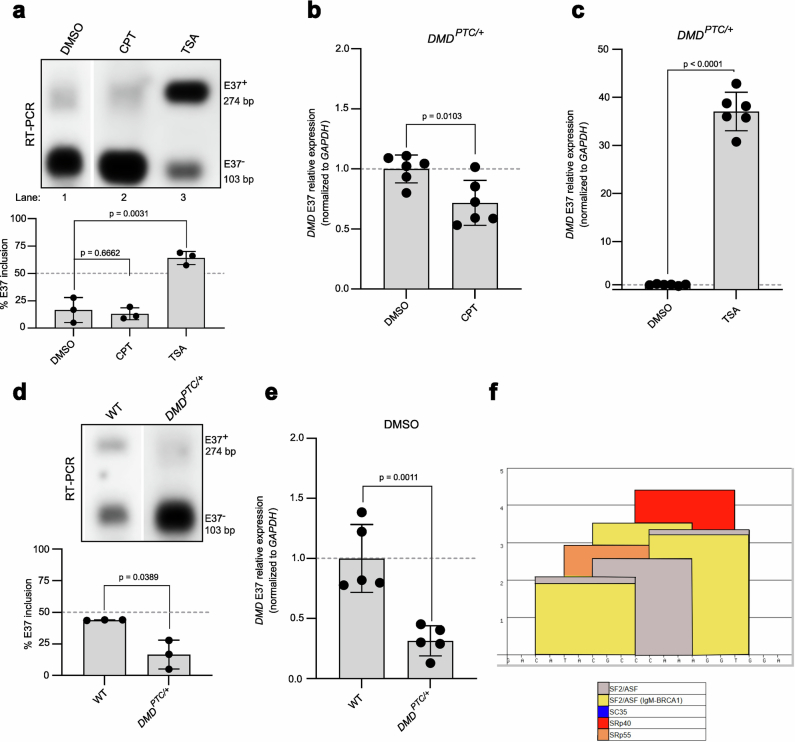
*DMD* E37 skipping in *DMD*^*PTC/+*^ cells. **a**, Effects on endogenous *DMD* E37 alternative splicing in *DMD*^*PTC/+*^ cells after treatment with CPT or TSA. Bars display means ± s.d. of the percentage of the intensity of the band containing E37 over the sum of intensity of the bands containing E37 and the skipped isoform (E37^-^) (n = 3 biologically independent samples). **b**,**c**, qPCR analysis of *DMD* E37 mRNA levels in *DMD*^*PTC/+*^ cells after treatment with DMSO, CPT (**b**), or TSA (**c**) (n = 6 biologically independent samples). **d**,**e**, *DMD* E37 skipping occurs more frequently in *DMD*^*PTC/+*^ cells than in WT cells (n = 3 (**d**) and 5 (**e**) biologically independent samples). **f**, Graphic output from ESEfinder showing colour bars, where the height of each bar indicates the score value, and its width represents its position along the deleted 20 nt sequence. Data are normalized to WT and are mean ± s.d.; a two-tailed Student’s *t*-test was used to calculate p values. Ct values included in Supplementary Table 5. Source Data

The ability to induce the inclusion of *DMD* E37 at will makes it a promising exon in which to introduce a frameshift or nonsense mutation that would lead to the decay of the *DMD* transcript. Therefore, we targeted E37 in HEK293T cells using a guide RNA with a high fidelity Cas9 and generated a heterozygous *DMD* line, *DMD*^*PTC/+*^, that carries a 20-nt deletion in E37, which results in a PTC positioned 118 nt upstream of its 3′ end (Fig. 1b). We measured the effect of CPT and TSA treatment on *DMD* E37 splicing in these cells, and found that the impact of elongation rate on *DMD* E37 inclusion was similar to that previously observed in WT cells (Extended Data Fig. 2a–c). We also observed that *DMD* E37 skipping happened more frequently in *DMD*^*PTC/+*^ cells than in WT cells in control conditions (Extended Data Fig. 2d,e), an observation consistent with previous reports showing that frameshift indels can lead to the skipping of affected in-frame exons, with the resulting transcripts escaping from nonsense-mediated mRNA decay (NMD)^34,35^. We then used the splicing-factor binding-site prediction tool, ESEfinder^36^, and found that the deleted 20-nt sequence in E37 was indeed predicted to harbour many splicing enhancers (Extended Data Fig. 2f), potentially explaining the more frequent skipping of E37 in *DMD*^*PTC/+*^ cells compared with WT.

## *DMD* E37^PTC^ inclusion trigger*s UTRN* upregulation

**Extended Data Fig. 3:**
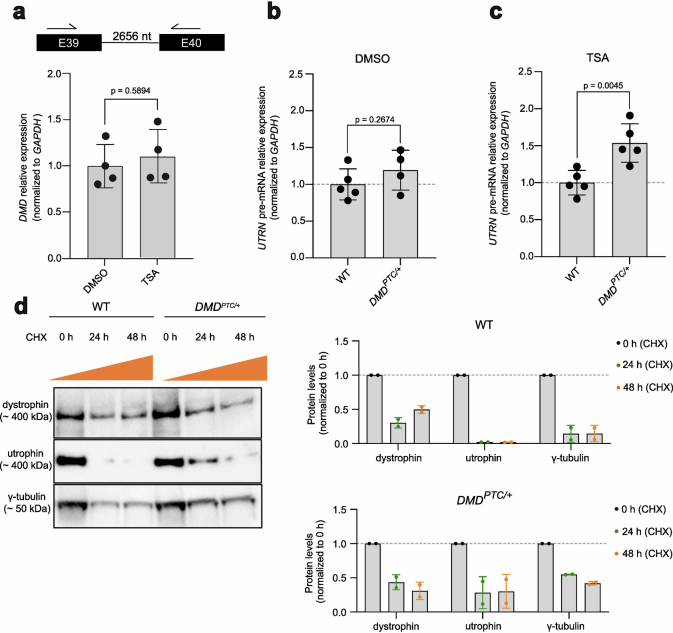
*UTRN* upregulation in *DMD*^*PTC/+*^ cells is due to increased transcription. **a**, qPCR analysis of *DMD* mRNA levels in WT cells after treatment with DMSO or TSA (n = 4 biologically independent samples). Black half-arrows indicate primers used. **b**,**c**, *UTRN* pre-mRNA levels are increased upon inclusion of the PTC – containing exon. qPCR analysis of *UTRN* pre-mRNA levels in WT and *DMD*^*PTC/+*^ cells after treatment with DMSO (**b**) or TSA (**c**) (n = 4 (**b**) and 5 (**c**) biologically independent samples). **d**, Western blot analysis and protein quantification showing dystrophin and utrophin levels upon treatment with 100 µg of cycloheximide (CHX) for 0, 24 and 48 h (n = 2 biologically independent samples). Data are normalized to WT and are mean ± s.d.; a two-tailed Student’s *t*-test was used to calculate p values. Ct values included in Supplementary Table 5. Source Data

To determine whether *DMD* mutant mRNA decay was taking place upon inclusion of the PTC-containing exon, we measured the mRNA levels of a region of the *DMD* transcript where the inclusion of exons was not affected by TSA treatment (that is, *DMD* E39-40) (Extended Data Fig. 3a). In control conditions, *DMD* expression levels were similar between WT and *DMD*^*PTC/+*^ cells (Fig. 1c), whereas they were significantly reduced in *DMD*^*PTC/+*^ cells compared with WT when we induced the inclusion of the PTC-containing exon (Fig. 1d), which is consistent with PTC bearing transcripts being subjected to NMD^37,38^. Given that the *mdx* mutant mouse, which harbours a nonsense mutation in *Dmd*, displays increased expression of utrophin mRNA and protein^12–14^, we then wanted to test whether utrophin mRNA and protein levels would be similarly upregulated when inducing the inclusion of the PTC-containing E37 in *DMD*^*PTC/+*^ cells. In contrast to control conditions, in which there are no changes in *UTRN* mRNA levels between WT and *DMD*^*PTC/+*^ cells (Fig. 1e), we observed an increase in *UTRN* expression levels in *DMD*^*PTC/+*^ cells compared with WT when treated with 1 µM TSA for 24 h (Fig. 1f). The fold change of this *UTRN* upregulation was around 1.5–2, which is similar to that reported in *mdx* mutant mice^39^. To investigate the cause of this increase in *UTRN* mRNA levels, we measured its precursor mRNA (pre-mRNA) levels, and found that they were also increased upon inclusion of the PTC-containing exon (Extended Data Fig. 3b,c), indicating that *UTRN* upregulation is due to increased transcription. We then investigated whether these changes in *DMD* and *UTRN* mRNA levels resulted in protein level changes. We observed an upregulation of utrophin protein despite no obvious loss of dystrophin protein (Fig. 1g,h), the latter most probably due to a long protein half-life as previously observed in vivo^40^. To evaluate the half-life of dystrophin and utrophin proteins, we treated both WT and *DMD*^*PTC/+*^ cells with the protein translation inhibitor cycloheximide for 24 and 48 h. We found that whereas utrophin levels were severely reduced at 24 h and almost absent at 48 h, dystrophin levels were reduced but still present at 48 h (Extended Data Fig. 3d). Altogether, these findings suggest that *UTRN* upregulation is not due to the loss of dystrophin protein but to RNA feedback loops involving *DMD* mutant mRNA decay. They also show that increased *UTRN* mRNA levels lead to increased utrophin protein levels.

## *DMD* mRNA decay precedes *UTRN* upregulation

**Extended Data Fig. 4:**
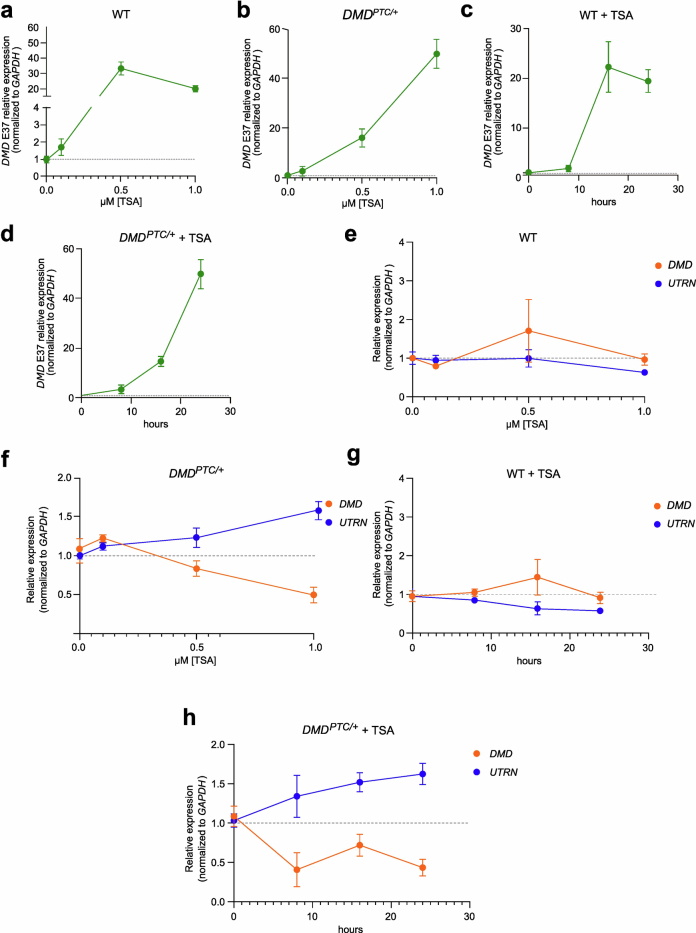
*UTRN* upregulation in *DMD*^*PTC/+*^ cells occurs in a TSA dose and time dependent manner. **a**-**d**, *DMD* E37 inclusion by TSA treatment is dose and time dependent. Effect on *DMD* E37 expression, as assessed by qPCR, in WT (**a, c**) and *DMD*^*PTC/+*^ (**b, d**) cells after treatment with 0.1, 0.5, or 1 µM TSA for 24 h (**a**, **b**), and after treatment with 1 µM TSA for 8, 16, or 24 h (**c**, **d**). **e**,**f**, *UTRN* mRNA levels increase in a TSA dose-dependent manner. qPCR analysis of *DMD* and *UTRN* mRNA levels in WT (**e**) and *DMD*^*PTC/+*^ (**f**) cells after treatment with 0.1, 0.5, or 1 µM TSA for 24 h. **g,h**, *UTRN* mRNA levels increase in a TSA time-dependent manner. qPCR analysis of *DMD* and *UTRN* mRNA levels in WT (**g**) and *DMD*^*PTC/+*^ (**h**) cells after treatment with 1 µM TSA for 8, 16, or 24 h. Data are normalized to WT and are mean ± s.d.; a two-tailed Student’s *t*-test was used to calculate p values (n = 2 or 3 biologically independent samples). Ct values included in Supplementary Table 6. Source Data

**Extended Data Fig. 5:**
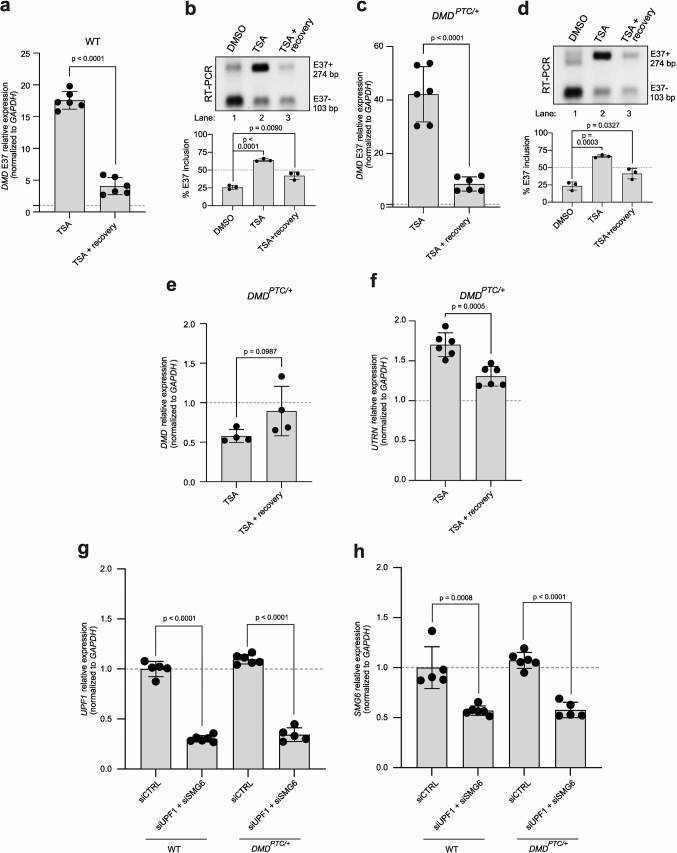
*UTRN* upregulation in *DMD*^*PTC/+*^ cells can be reversed by washing away TSA; UPF1 and SMG6 knockdown efficiency. **a**-**d**, Effects on *DMD* E37 expression and alternative splicing in WT (**a**, **b**) and *DMD*^*PTC/+*^ (**c**, **d**) cells after treatment with 1 µM TSA for 24 h or 1 µM TSA for 24 h followed by 48 h of recovery from TSA (TSA + recovery) (n = 6 (**a**, **c**) and 3 (**b**, **d**) biologically independent samples). Bars display means ± s.d. of the percentage of the intensity of the band containing E37 over the sum of intensity of the bands containing E37 and the skipped isoform (E37^−^). **e**,**f**, *UTRN* upregulation is reversed upon recovery from TSA. qPCR analysis of *DMD* (n = 4 biologically independent samples) (**e**) and *UTRN* (n = 6 biologically independent samples) (**f**) mRNA levels after treatment with 1 µM TSA for 24 h or 1 µM TSA for 24 h followed by 48 h of recovery from TSA (TSA + recovery). Data are normalized to WT and are mean ± s.d.; a two-tailed Student’s *t*-test was used to calculate p values. **g**,**h**, qPCR analysis of *UPF1* (**g**) and *SMG6* (**h**) mRNA levels after siRNA-mediated *UPF1* and *SMG6* knockdown in WT and *DMD*^*PTC/+*^ cells treated with TSA (n = 5 biologically independent samples). Data are normalized to WT treated with TSA and siCTRL and are mean ± s.d.; a two-tailed Student’s *t*-test was used to calculate p values. Ct values included in Supplementary Table 6. Source Data

The effect of TSA treatment on *DMD* E37 inclusion is dose and time dependent in both WT and *DMD*^*PTC/+*^ cells (Extended Data Fig. 4a–d). Increasing the concentration of TSA led to a more pronounced decrease in *DMD* mRNA levels and a more pronounced increase in *UTRN* mRNA levels (Extended Data Fig. 4e,f). In terms of time dependency, after 8 h of treatment with TSA, a significant decrease in *DMD* mRNA levels was already present in *DMD*^*PTC/+*^ cells compared with WT, whereas *UTRN* mRNA levels were only slightly altered at this time; however, *UTRN* mRNA levels were significantly upregulated (approximately 1.5-fold) after 16 h of treatment (Extended Data Fig. 4g,h). Together, these findings suggest that changes in *UTRN* mRNA levels are caused by *DMD* mutant mRNA decay. We could also reverse the effects of TSA by washing it away and letting the cells recover for 48 h. Recovery from TSA led to a reduction in *DMD* E37 inclusion levels (Extended Data Fig. 5a–d) as well as a normalization in *DMD* and *UTRN* mRNA levels (Extended Data Fig. 5e,f).

## Blocking NMD normalizes *UTRN* upregulation

**Fig. 2:**
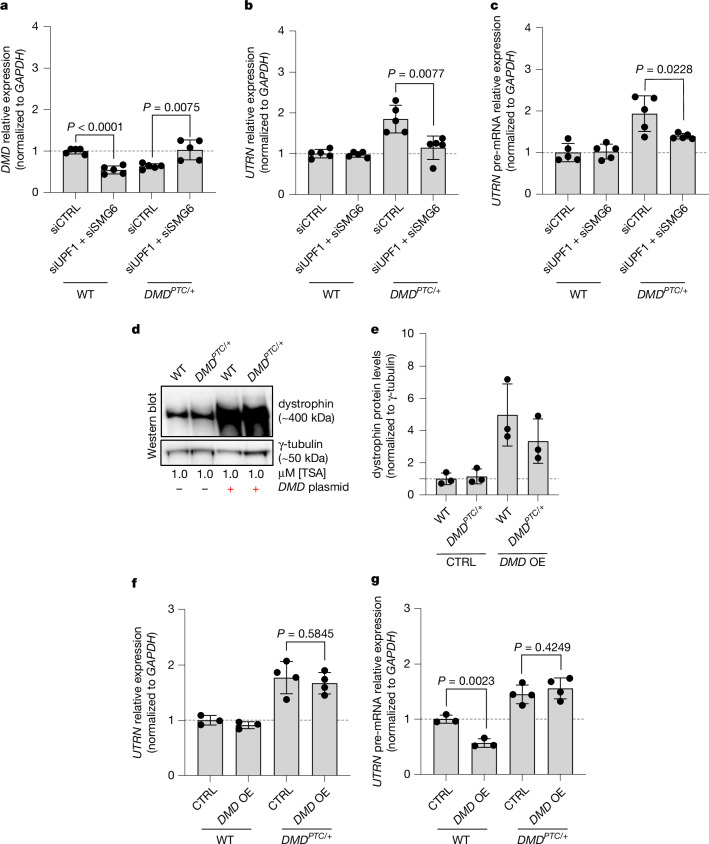
Blocking NMD results in the reversal of *UTRN* upregulation, whereas overexpressing *DMD* does not. **a**–**c**, Blocking NMD results in the normalization of *DMD* expression as well as a 62% loss of *UTRN* upregulation at the mRNA level and a 70% loss at the pre-mRNA level. qPCR analysis of *DMD* mRNA (**a**), *UTRN* mRNA (**b**) and *UTRN* pre-mRNA (**c**) levels after siRNA-mediated knockdown of UPF1 and SMG6 in WT and *DMD*^*PTC/+*^ cells treated with TSA (*n* = 5 biologically independent samples); siCTRL, scrambled control. **d**–**f**, Overexpression (OE) of dystrophin protein does not alter the upregulation of *UTRN* caused by the inclusion of the *DMD* E37^PTC/+^. **d**,**e**, Western blot analysis (**d**) and protein quantification (**e**) showing the OE of dystrophin protein following transfection of a *DMD* overexpression plasmid (*n* = 3 biologically independent samples). **f**,**g**, qPCR analysis of *UTRN* mRNA (**f**) and *UTRN* pre-mRNA (**g**) levels in WT and *DMD*^*PTC/+*^ HEK293T cells transfected with an empty vector or the *DMD* overexpression plasmid, followed by addition of 1 µM TSA for 24 h (*n* = 4 biologically independent samples). Data are normalized to WT and are mean ± s.d.; a two-tailed Student’s *t*-test was used to calculate *P* values. Ct values are included in Supplementary Table 5. Source Data

To investigate the role of the NMD surveillance machinery in *DMD*^*PTC/+*^ cells treated with TSA, we knocked down UPF1 and SMG6—two key NMD proteins^37,38^ (Extended Data Fig. 5g,h). Blocking NMD in *DMD*^*PTC/+*^ cells led to an increase in *DMD* mutant mRNA levels (Fig. 2a) as well as a loss of *UTRN* upregulation at the mRNA (Fig. 2b) and pre-mRNA (Fig. 2c) levels. Incidentally, although blocking NMD in WT cells had no effect on *UTRN* mRNA or pre-mRNA levels compared with a scrambled control (Fig. 2b,c), it did lead to a significant reduction in *DMD* mRNA levels (Fig. 2a). Together, the data from the *DMD*^*PTC/+*^ cells indicate that *DMD* mutant mRNA decay is required to trigger *UTRN* upregulation, which led us to explore whether TA, which is triggered by mutant mRNA decay and not protein loss^6,8^, was taking place.

## *DMD* OE does not reduce *UTRN* upregulation

**Extended Data Fig. 6:**
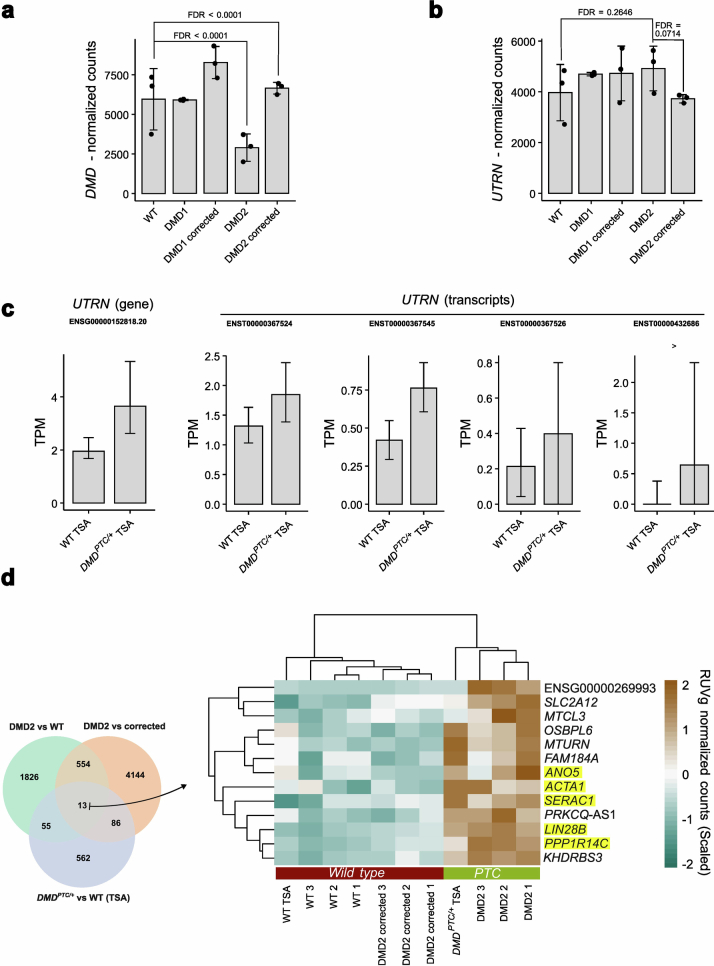
Other genes affected by *DMD* nonsense-mediated mRNA decay. **a**,**b**, Normalized *DMD* (**a**) and *UTRN* (**b**) mRNA counts from RNA-seq analysis of WT, DMD1, DMD2, DMD1-corrected, and DMD2-corrected iPSCs differentiated into myotubes^41^ (n = 3 biologically independent samples). **c**, Bootstrapped TPM RNA-seq averages and 95% confidence limits (see methods) for the *UTRN* gene and its isoforms in WT and *DMD*^*PTC/+*^ HEK293T cells treated with TSA (n = 1 biological sample). **d**, RNA-seq Venn diagram (left) showing upregulated genes in DMD2 compared with WT and with DMD2-corrected, as well as *DMD*^*PTC/+*^ HEK293T cells compared with WT. Heatmap (right) shows batch-normalized combined read counts from both experiments, depicting the upregulated intersection. TPM: transcripts per million; CI95: 95% confidence interval; FDR: false discovery rate. Source Data

To test the hypothesis that *UTRN* upregulation in *DMD*^*PTC/+*^ cells is caused by TA, we first overexpressed *DMD* in *DMD*^*PTC/+*^ cells treated with TSA. If *UTRN* upregulation in *DMD*^*PTC/+*^ cells is due to a protein feedback effect, dystrophin overexpression should normalize *UTRN* mRNA levels. However, if *UTRN* upregulation is due to TA, dystrophin overexpression should not affect it^8^. In line with the hypothesis that *UTRN* upregulation in *DMD*^*PTC/+*^ cells is caused by TA, dystrophin protein overexpression in these cells did not affect *UTRN* mRNA or pre-mRNA upregulation (Fig. 2d–g). Thus, *DMD* mutant mRNA decay, and not the loss of dystrophin protein, seems to play a key role in *UTRN* upregulation in *DMD*^*PTC/+*^ cells. We then sought to identify whether other genes were also affected by *DMD* mutant mRNA decay, potentially through TA. Therefore, we reanalysed a published RNA sequencing (RNA-seq) dataset from specific induced pluripotent stem (iPS) cells from DMD patients differentiated into myotubes^41^. These iPS cells were derived from two different patients, one with an E45 deletion (DMD1) and the other with an E51 deletion (DMD2), and the mutations were corrected through knock-ins. We focused our analysis on the DMD2 samples for several reasons including the upregulation of *UTRN* in mutant cells, which was reduced after mutation correction (Extended Data Fig. 6a,b and Methods). Given that these DMD2 cells seem to display TA, we proceeded to search for other potential adapting genes (that is, genes upregulated due to *DMD* mutant mRNA decay) and validated them by determining the transcriptome of *DMD*^*PTC/+*^ HEK293T cells treated with TSA, which exhibit *UTRN* upregulation by RNA-seq analysis as well (Extended Data Fig. 6c). We found that some of the commonly upregulated genes are involved, directly or indirectly, in muscle function, such as *ANO5* (related to limb girdle muscular dystrophy type 2L), *ACTA1* (related to congenital myopathy), *SERAC1* (related to muscular dystonia), *LIN28B* (with functions in muscle development) and *PPP1R14C* (involved in muscle contraction) (Extended Data Fig. 6d), suggesting that more genes, beyond *UTRN*, may compensate for the loss of dystrophin via their upregulation. We found in another published RNA-seq dataset^14^ that *Acta1* also showed a trend towards upregulation, although not significant, in *mdx* mutant skeletal muscle compared with WT.

## *DMD*^*PTC*^ minigenes trigger *UTRN* upregulation

**Fig. 3:**
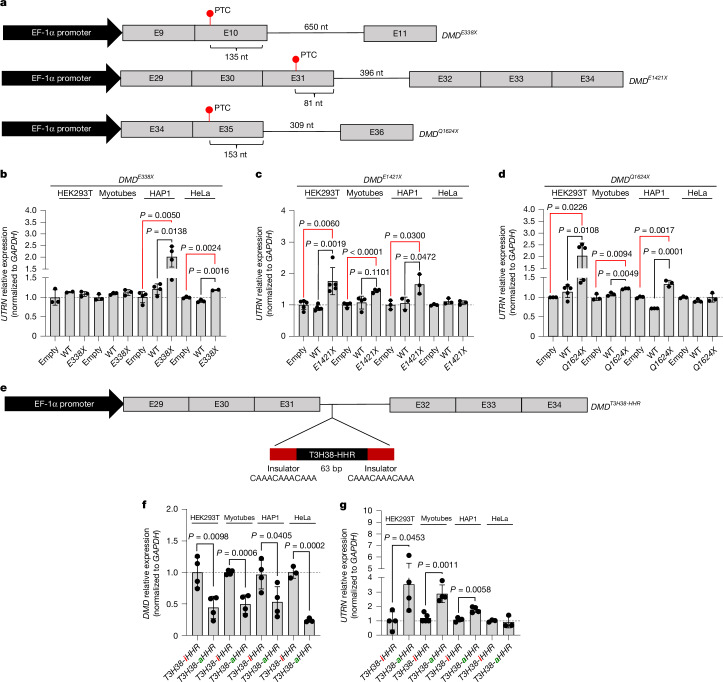
*UTRN* upregulation induced by *DMD*^*PTC*^ minigenes and by a *DMD*^*WT*^ minigene containing a self-cleaving ribozyme. **a**, Schematic illustration of the *DMD*^*PTC*^ minigenes. **b**–**d**, OE of the *DMD*^*PTC*^ minigenes in WT cells results in *UTRN* upregulation. qPCR analysis of *UTRN* mRNA levels in WT HEK293T cells, myotubes, HAP1 cells and HeLa cells transfected with an empty vector, the *DMD*^*WT*^ minigene or the *DMD*^*E338X*^ (**b**), *DMD*^*E1421X*^ (**c**) or *DMD*^*Q1624X*^ (**d**) minigene (*n* = 3 biologically independent samples). **e**, Schematic illustration of the *DMD*^*WT*^ minigene containing a self-cleaving ribozyme (T3H38-HHR) inserted in intron 31, flanked by two insulator sequences. Both an inactive and an active version of the ribozyme were used. **f**,**g**, OE of the *DMD*^*WT*^ minigene containing T3H38-HHR in WT cells results in *UTRN* upregulation. qPCR analysis of *DMD* (exogenous) (**f**) and *UTRN* (**g**) mRNA levels in WT HEK293T cells, myotubes, HAP1 cells and HeLa cells transfected with the *DMD*^*WT*^ minigene with an inactive ribozyme (T3H38-iHHR) or with an active ribozyme (T3H38-aHHR) (*n* = 4 biologically independent samples). Data are normalized to WT transfected with the empty vector (**b**–**d**) or with *DMD*^*T3H38-iHHR*^ (**f**,**g**) and are mean ± s.d.; a two-tailed Student’s *t*-test was used to calculate *P* values; *P* values shown only when significant. Ct values are included in Supplementary Table 5. Source Data

**Extended Data Fig. 7:**
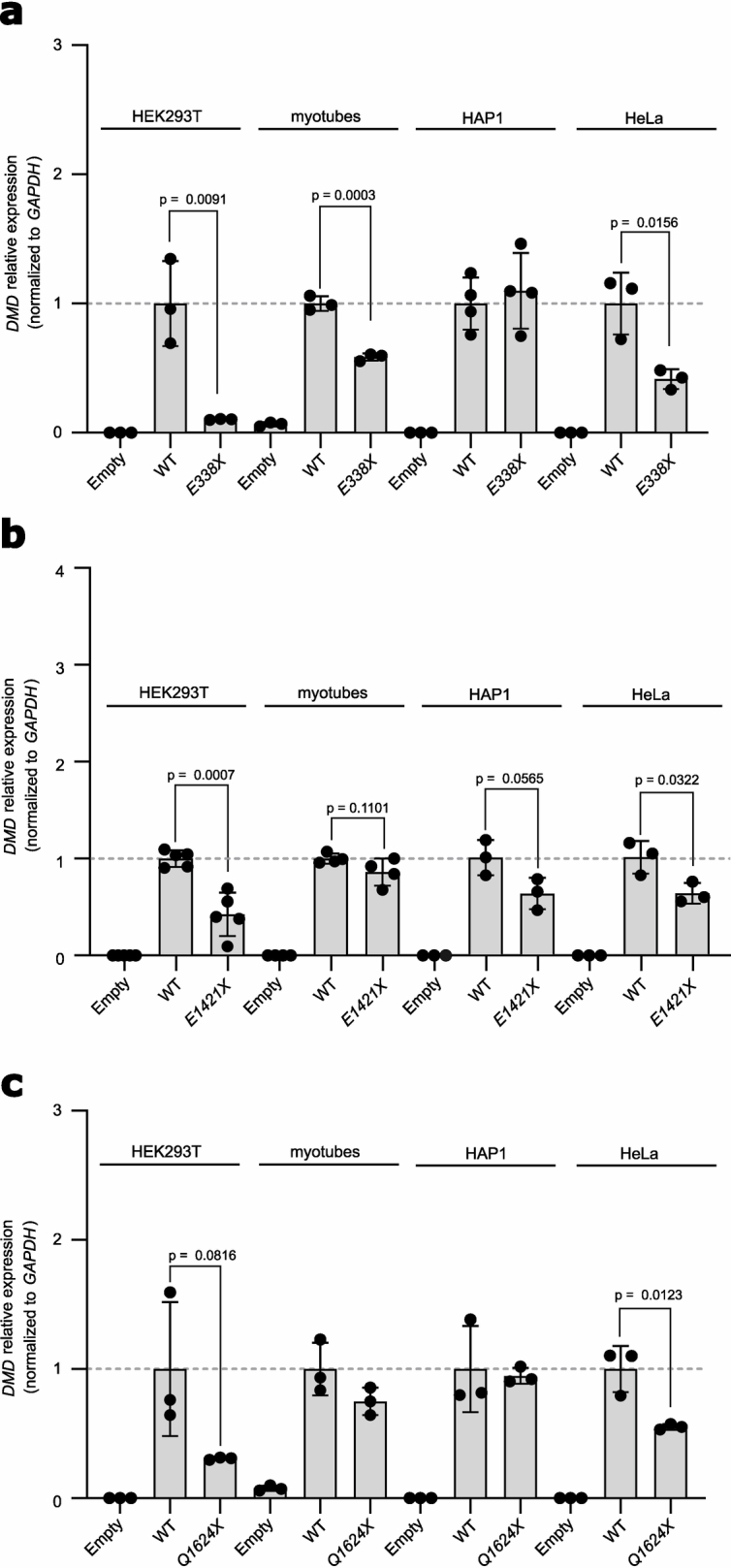
Exogenous *DMD* mRNA levels are lower in cells transfected with *DMD*^*PTC*^ minigenes than with *DMD*^*WT*^ minigenes. **a**-**c**, qPCR analysis of exogenous *DMD* mRNA levels in WT HEK293T cells, myotubes, HAP1 cells, and HeLa cells transfected with an empty vector, the *DMD* WT minigene, or the *DMD* E338X (**a**), E1421X (**b**), or Q1624X (**c**) minigene (n = 3 biologically independent samples). Data are normalized to WT cells transfected with an empty vector and are mean ± s.d.; a two-tailed Student’s *t*-test was used to calculate p values. Ct values included in Supplementary Table 5. Source Data

To further test the hypothesis that TA can trigger *UTRN* upregulation and investigate whether one could induce *UTRN* upregulation in WT cells, we generated three different *DMD* minigenes, one consisting of E9 to E11 with intron 10, the second of E29 to E34 with intron 31, and the third of E34 to E36 with intron 35, hereafter referred to as the *DMD*^*WT*^ minigenes (Fig. 3a). We then introduced nonsense mutations in E10 (E338X), E31 (E1421X) and E35 (Q1624X), respectively, to obtain the *DMD*^*PTC*^ minigenes. We transfected the *DMD*^*WT*^ and *DMD*^*PTC*^ minigenes, or an empty vector—consisting of the plasmid backbone in which the minigenes were generated—into WT HEK293T, HAP1 and HeLa cells as well as into myotubes. For most cell lines, we observed lower exogenous *DMD* mRNA levels following transfection with the *DMD*^*PTC*^ minigenes compared with transfection with the *DMD*^*WT*^ minigenes (Extended Data Fig. 7a–c), suggesting that degradation of the *DMD* transcripts encoded by the PTC minigenes is taking place. For the *DMD*^*E1421X*^ and *DMD*^*Q1624X*^ constructs, we observed increased *UTRN* mRNA levels upon transfection of the *DMD*^*PTC*^ minigenes, but not of the *DMD*^*WT*^ minigenes, compared with transfections with the empty vector (Fig. 3c,d), in all cell lines except for HeLa cells. For the *DMD*^*E338X*^ minigene, increased *UTRN* mRNA levels were observed only in HAP1 and HeLa cells (Fig. 3b). Altogether, these findings further indicate that *DMD* mutant mRNA decay can trigger *UTRN* upregulation; they also suggest that the *UTRN* upregulation response varies among different cell types and depending on the location of the PTC.

## A *DMD*^*HHR*^ minigene triggers *UTRN* upregulation

We then investigated another approach to degrade *DMD* RNA without affecting dystrophin protein levels. To this end, we inserted a modified variant of the *Schistosoma mansoni* hammerhead ribozyme (HHR), hereafter referred to as T3H38-HHR (ref. ^42^), into the intron of a *DMD*^*WT*^ minigene that consists of E29 to E34 with intron 31 (Fig. 3e). HHRs are small self-cleaving ribozymes that fold into defined structures and catalyse their own cleavage^43^. A single nucleotide substitution alters T3H38-HHR from its catalytically active form (denoted T3H38-aHHR) to an inactive form (denoted T3H38-iHHR)^42^, which we used as a control. WT HEK293T, HAP1 and HeLa cells as well as myotubes transfected with the *DMD*^*T3H38-aHHR*^ construct displayed a decrease in exogenous *DMD* mRNA levels compared with cells transfected with the *DMD*^*T3H38-iHHR*^ construct (Fig. 3f). Furthermore, *UTRN* mRNA levels were increased in most cell types transfected with the *DMD*^*T3H38-aHHR*^ construct compared with cells transfected with the *DMD*^*T3H38-iHHR*^ construct (Fig. 3g). In HeLa cells, we observed no upregulation of *UTRN* mRNA levels upon transfection with the *DMD*^*T3H38-aHHR*^ construct compared with the *DMD*^*T3H38-iHHR*^ construct (Fig. 3g), in line with the results obtained using the *DMD*^*E1421X*^ minigene, the PTC counterpart (Fig. 3c). These findings with the PTC and ribozyme minigenes, which cause an increase in *UTRN* mRNA levels in the presence of *DMD* minigene mRNA/pre-mRNA decay despite no loss of endogenous dystrophin protein, further indicate that *DMD* mRNA decay is the main trigger for *UTRN* upregulation in *DMD*^*PTC/+*^ cells, placing TA as the mechanism behind *UTRN* upregulation in some DMD patients.

## *DMD*^*PTC*^ myotubes display *UTRN* upregulation

**Fig. 4:**
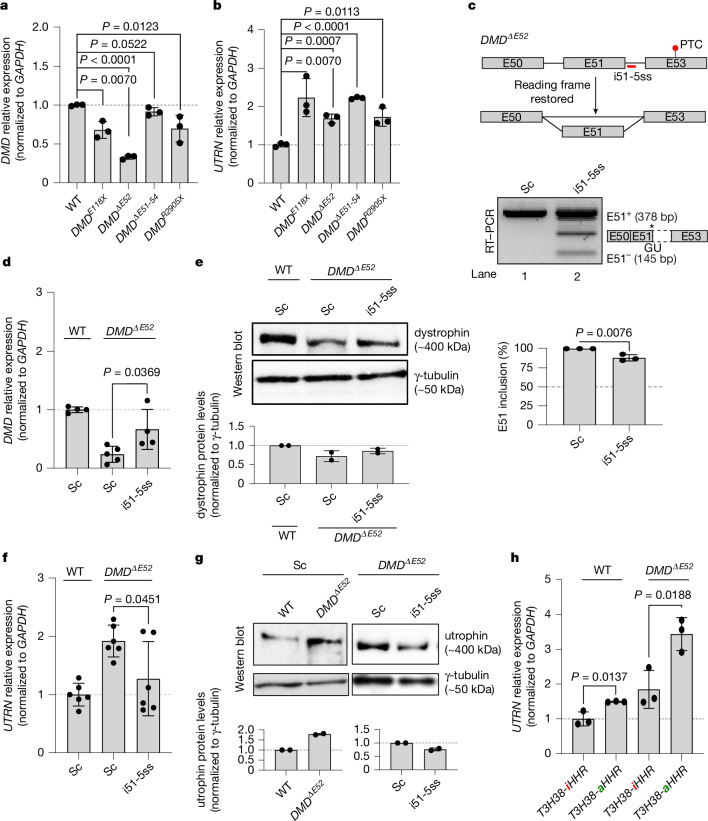
Myotubes from *DMD*^*PTC*^ patients display *UTRN* upregulation, and restoring the *DMD* reading frame in *DMD*^*ΔE52*^ myotubes reduces this upregulation. **a**,**b**, qPCR analysis of *DMD* (**a**) and *UTRN* (**b**) mRNA levels in myotubes derived from DMD patients compared with myotubes derived from healthy control participants (*n* = 3 biologically independent samples). **c**, Schematic of the binding site of the i51-5ss ASO and RT–PCR showing *DMD* E51 skipping upon transfection in *DMD*^*ΔE52*^ myotubes. Bars display means ± s.d. of the percentage of the intensity of the E51-containing band over the sum of intensity of the bands containing E51 and the skipped isoform (E51^−^) (*n* = 3 biologically independent samples). The asterisk * denotes a cryptic splice site. **d**, Restoring the reading frame in *DMD*^*ΔE52*^ myotubes results in a 175% increase in *DMD* mRNA levels. qPCR analysis of *DMD* mRNA levels in WT and *DMD*^*ΔE52*^ myotubes after transfection with a scrambled (Sc) control ASO or i51-5ss (*n* = 4 biologically independent samples). **e**, Western blot analysis and protein quantification showing dystrophin levels in WT and *DMD*^*ΔE5*^ myotubes after transfection with the Sc ASO or i51-5ss (*n* = 2 biologically independent samples). **f**, Restoring the reading frame in *DMD*^*ΔE52*^ myotubes results in a 33.85% decrease in *UTRN* mRNA levels. qPCR analysis of *UTRN* mRNA levels in WT and *DMD*^*ΔE52*^ myotubes after transfection with the Sc ASO or i51-5ss (*n* = 6 biologically independent samples). **g**, Western blot analysis and protein quantification showing utrophin levels in WT and *DMD*^*ΔE52*^ myotubes after transfection with the Sc ASO or i51-5ss (*n* = 2 biologically independent samples). **h**, qPCR analysis of *UTRN* mRNA levels in WT and *DMD*^*ΔE52*^ myotubes transfected with the *DMD*^*T3H38-iHHR*^ or *DMD*^*T3H38-aHHR*^ minigene (*n* = 3 biologically independent samples). Data are normalized to WT or to the Sc ASO samples, and are mean ± s.d.; two-tailed Student’s *t*-test used to calculate *P* values. Ct values are in Supplementary Table 5. Source Data

**Extended Data Fig. 8:**
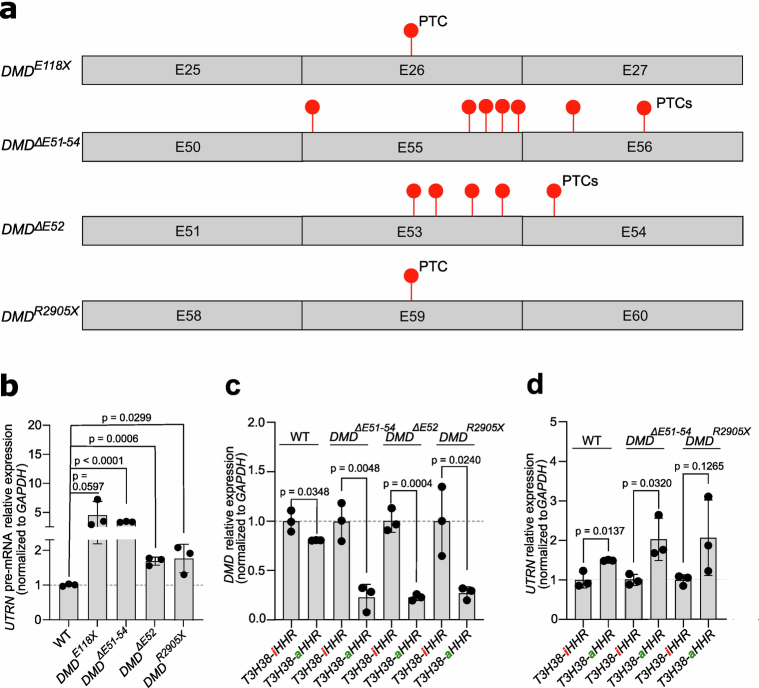
*UTRN* upregulation in *DMD*^*PTC*^ patient-derived myotubes is due to increased transcription; and a *DMD* minigene containing a self-cleaving ribozyme enhances *UTRN* upregulation in *DMD*^*PTC*^ myotubes. **a**, Schematic illustration of the *DMD*^*PTC*^ alleles for the DMD patient-derived myotubes; red indicates the PTCs. **b**, *UTRN* pre-mRNA levels are increased in DMD patient-derived myotubes. qPCR analysis of *UTRN* pre-mRNA levels in the DMD patient-derived myotubes (n = 3 biologically independent replicates). **c**, qPCR analysis of exogenous *DMD* mRNA levels in WT, *DMD*^*ΔE51-54*^, *DMD*^*ΔE52*^, and *DMD*^*R2905X*^ patient-derived myotubes transfected with the *DMD*^*T3H38-iHHR*^ or *DMD*^*T3H38-aHHR*^ ribozyme minigenes (n = 3 biologically independent samples). **d**, qPCR analysis of *UTRN* mRNA levels in WT, *DMD*^*ΔE51-54*^, and *DMD*^*R2905X*^ patient-derived myotubes transfected with the *DMD*^*T3H38-iHHR*^ or *DMD*^*T3H38-aHHR*^ ribozyme minigenes (n = 3 biologically independent samples). Data are normalized to WT (**b**) or WT transfected with the *DMD*^*T3H38-iHHR*^ ribozyme minigene **(c**, **d**) and are mean ± s.d.; a two-tailed Student’s *t*-test was used to calculate p values. Ct values included in Supplementary Table 5. Source Data

To place these findings in a disease context, we first measured *DMD* and *UTRN* mRNA levels in myotubes derived from four DMD patients, each carrying a different *DMD* lesion predicted to lead to PTCs: a nonsense mutation in E26 (*DMD*^*E118X*^), a deletion spanning from E51 to E54 (*DMD*^*ΔE51-54*^), an E52 deletion (*DMD*^*ΔE52*^) and a nonsense mutation in E59 (*DMD*^*R2905X*^) (Extended Data Fig. 8a). We observed reduced *DMD* mRNA levels and increased *UTRN* mRNA levels in all DMD derived myotubes compared with myotubes derived from a healthy control (Fig. 4a,b). Furthermore, as previously observed in HEK293T cells (Extended Data Fig. 3b,c), we also found increased *UTRN* pre-mRNA levels in myotubes derived from the DMD patients compared with myotubes derived from a healthy control participant (Extended Data Fig. 8b), indicating that *UTRN* upregulation in these cases is also due to increased transcription.

## *UTRN* expression in *DMD*^*ΔE52*^ myotubes

We then sought to restore the *DMD* reading frame in *DMD*^*ΔE52*^ myotubes through exon skipping, a current treatment for certain DMD patients with specific mutations in *DMD*. For example, eteplirsen is an FDA-approved ASO designed to treat DMD patients with a disrupted reading frame that can be restored by the skipping of exon 51 (refs. ^44,45^), which allows the production of an internally deleted but partially functional dystrophin protein. We therefore designed an ASO similar to eteplirsen that promotes exon 51 (i51-5ss) skipping similar to eteplirsen and introduced it in *DMD*^*ΔE52*^ myotubes (Fig. 4c). Notably, we found that this ASO not only led to increased *DMD* mRNA levels in these *DMD* mutant myotubes (Fig. 4d,e), but that it also reduced utrophin upregulation (Fig. 4f,g), thereby potentially counteracting some of the beneficial effects of restoring the reading frame. We then decided to try to enhance *UTRN* upregulation rather than block it in these *DMD*^*ΔE52*^ myotubes. Therefore, we took advantage of the *DMD*^*WT*^ minigene containing the self-cleaving ribozyme (*DMD*^*T3H38-HHR*^) to determine whether *UTRN* mRNA levels could be further upregulated in *DMD*^*ΔE52*^ myotubes. We indeed observed higher *UTRN* mRNA levels in *DMD*^*ΔE52*^ myotubes transfected with the active ribozyme compared with those transfected with the inactive ribozyme (Fig. 4h). We also observed increased *UTRN* mRNA levels in other myotubes derived from DMD patients transfected with the active ribozyme compared with those transfected with the inactive ribozyme (Extended Data Fig. 8c,d).

## Splice-switching ASOs can trigger *UTRN* upregulation

**Fig. 5:**
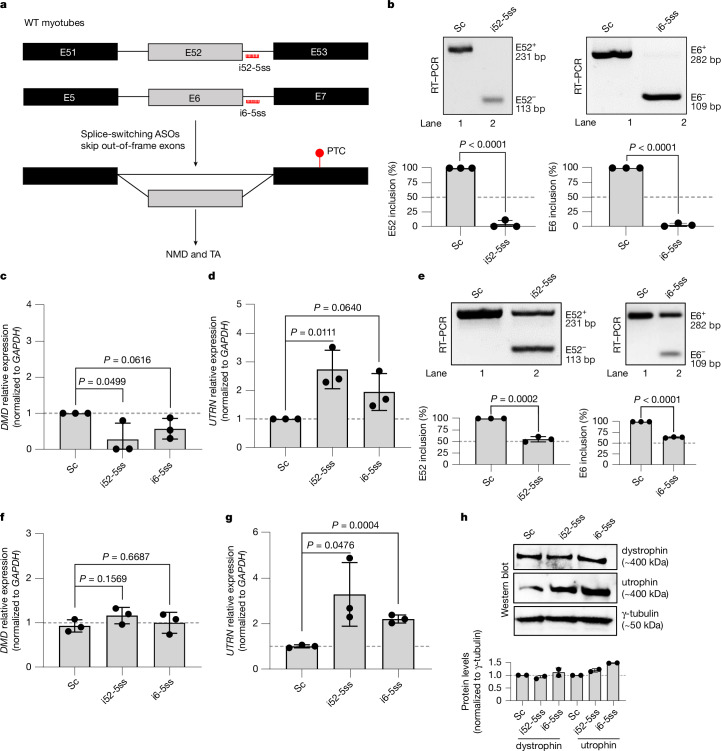
Introducing PTCs in *DMD* using splice-switching ASOs triggers *UTRN* upregulation. **a**, Schematic illustration of splice-switching ASOs disrupting the *DMD* reading frame by inducing the skipping of the out-of-frame exons E52 and E6, thereby leading to the introduction of PTCs and TA. **b**, RT–PCR showing *DMD* E52 or E6 full skipping upon i52-5ss or i6-5ss transfection in WT myotubes. Bars display means ± s.d. of the percentage of the intensity of the bands containing E52 or E6 over the sum of intensity of the bands containing E52 or E6 and the skipped isoform (E52^−^ or E6^−^) (*n* = 3 biologically independent samples). **c**,**d**, Skipping the out-of-frame *DMD* exons E52 or E6 triggers *UTRN* upregulation. qPCR analysis of *DMD* (**c**) and *UTRN* (**d**) mRNA levels in WT myotubes after transfection with the Sc ASO, i52-5ss or i6-5ss (*n* = 3 biologically independent samples). **e**–**g**, Graphs show the results from the same experiments as in **b** (**e**), **c** (**f**) and **d** (**g**) but with the ASO transfections inducing partial exon skipping (*n* = 3 biologically independent samples). Data are normalized to the Sc ASO samples and are mean ± s.d.; a two-tailed Student’s *t*-test was used to calculate *P* values. **h**, Western blot analysis and protein quantification showing dystrophin and utrophin levels in WT myotubes treated with the Sc ASO, i52-5ss or i6-5ss at concentrations inducing partial exon skipping (*n* = 2 biologically independent samples). Ct values are included in Supplementary Table 5. Source Data

**Extended Data Fig. 9:**
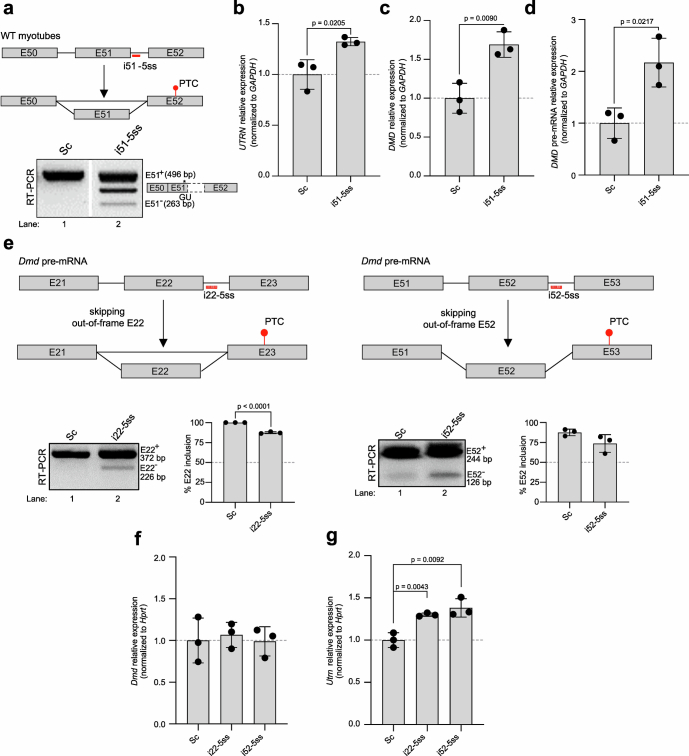
*UTRN*/*Utrn* upregulation triggered by splice-switching ASOs. **a**, RT-PCR showing *DMD* E51 skipping upon transfection of an ASO targeting the 5’ splice site of intron 51 (i51-5ss) in WT myotubes. Bars display means ± s.d. of percentage of the intensity of the band containing E51 over the sum of intensity of the bands containing E51 and the skipped isoform (E51^−^) (n = 3 biologically independent samples). *, cryptic splice site. **b**-**d**, qPCR analysis of *DMD* (**b**) and *UTRN* (**c**) mRNA levels, as well as *DMD* pre-mRNA levels (**d**), in WT myotubes after treatment with Sc or i51-5ss (n = 3 biologically independent samples). **e**, RT-PCR showing *Dmd* E22 and *Dmd* E52 skipping upon transfection of ASOs targeting the 5’ splice site of intron 22 (i22-5ss) or 52 (i52-5ss) in WT C2C12 cells. Bars display means ± s.d. of percentage of the intensity of the bands containing E22 or E52 over the sum of intensity of the bands containing E22 or E52 and the skipped isoform (E22^−^ or E51^−^) (n = 3 biologically independent samples). **f**,**g**, qPCR analysis of *Dmd* (**f**) and *Utrn* (**g**) mRNA levels in WT myotubes after treatment with Sc, i22-5ss, or i52-5ss (n = 3 biologically independent samples). Data are normalized to WT transfected with the Sc ASO and are mean ± s.d.; a two-tailed Student’s *t*-test was used to calculate p values. Ct values included in Supplementary Table 5. Source Data

In a converse approach to restoring the reading frame, we transfected WT myotubes with splice-switching ASOs designed to target the 5′ splice sites of two out-of-frame *DMD* exons (E6 (i6-5ss) and E52 (i52-5ss)), and thereby induce their skipping (Fig. 5a). We reasoned that skipping E6 or E52 should lead to the introduction of PTCs in the *DMD* transcript by disrupting the reading frame, and thereby trigger *UTRN* upregulation (Fig. 5a). We tested these ASOs in WT myotubes and observed an almost 100% efficient skipping of E6 and E52 (Fig. 5b) when compared with a scrambled control ASO (Sc). Skipping E6 or E52 triggered *DMD* mutant mRNA decay as well as *UTRN* upregulation (Fig. 5c,d). Next, we reasoned that if *UTRN* upregulation by means of splice-switching ASOs was mediated by TA and not protein loss, then partial exon skipping (that is, 25–50%) should also lead to *UTRN* upregulation without causing a severe reduction in *DMD* mRNA or protein levels. Partial exon skipping indeed led to *UTRN* upregulation (Fig. 5g,h) without causing a significant reduction in *DMD* mRNA (Fig. 5f) or protein (Fig. 5h) levels, probably due to the higher prevalence of the full-length isoform compared with the isoforms in which E6 or E52 is skipped (Fig. 5e). Furthermore, we also used the i51-5ss ASO, which we had used in Fig. 4c–g to restore the reading frame in *DMD*^*ΔE52*^ myotubes, to disrupt the reading frame in WT myotubes, as E51 is also an out-of-frame exon (Extended Data Fig. 9a). As expected, we observed *UTRN* upregulation (Extended Data Fig. 9b). We also observed an increase in *DMD* mRNA levels in WT myotubes transfected with the i51-5ss ASO compared with those transfected with the Sc ASO (Extended Data Fig. 9c), which could be explained by self-TA^8^ (that is, *DMD* mutant mRNA decay leads to the transcriptional upregulation of *DMD* itself). To further test whether self-TA could be occurring with this ASO, we measured *DMD* pre-mRNA levels and indeed observed their increase in WT myotubes transfected with the i51-5ss ASO compared with the Sc ASO (Extended Data Fig. 9d). We then decided to use this approach (that is, splice-switching ASOs that disrupt the reading frame) in the C2C12 mouse skeletal muscle cell line to determine whether *Utrn* upregulation could be observed upon inducing *Dmd* mRNA decay in mouse as in human. We used a splice-switching ASO targeting the out-of-frame *Dmd* E22, leading to PTCs in E23 (which incidentally is also the PTC-containing exon in the *mdx* mutant mouse^12^), as well as an ASO targeting the out-of-frame E52 (Extended Data Fig. 9e,g). We observed *Utrn* upregulation, despite no significant changes in *Dmd* mRNA levels, with both of these splice-switching ASOs compared with scrambled control (Extended Data Fig. 9f,g), indicating that in mouse as in human, TA can trigger *Utrn*/*UTRN* upregulation in *Dmd*/*DMD*^*PTC/+*^ cells.

## Discussion

**Extended Data Fig. 10:**
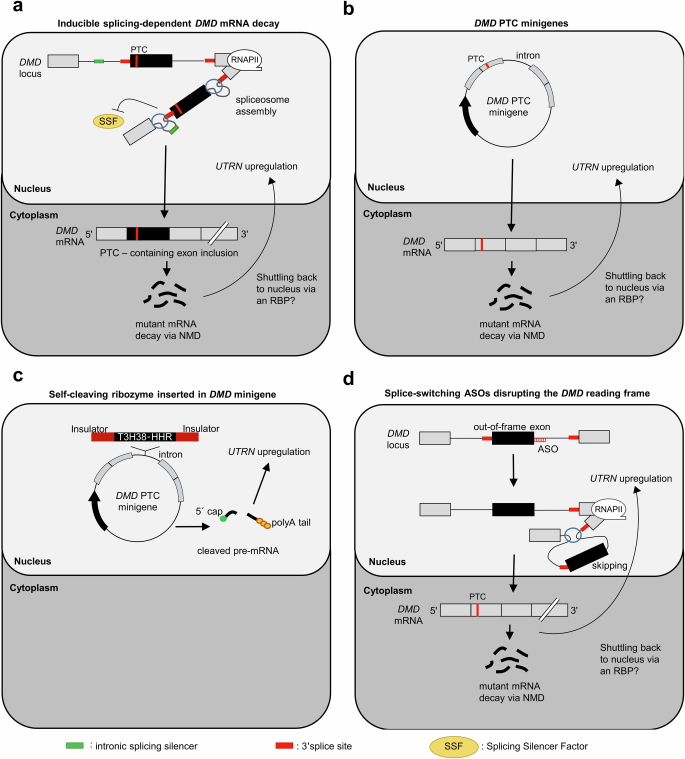
Transcriptional adaptation drives *UTRN* upregulation upon *DMD* RNA decay. Model summarizing the experimental approaches and data. **a**, The intronic sequence bound by a splicing silencer factor (SSF) is depicted by green boxes, the 3’ splice sites by red boxes, and the constitutive exons surrounding the alternatively spliced *DMD* E37 by grey boxes. The spliceosome is depicted by light blue circles. Promoting fast transcriptional elongation (presumably by preventing SSF from binding to the intronic splicing silencer) leads to the inclusion of E37, which contains a PTC, in the mRNA. The inclusion of the PTC-containing exon triggers NMD and TA-induced *UTRN* upregulation. **b**, *DMD*^*PTC*^ minigenes generate a PTC-containing mRNA that is degraded, leading to *UTRN* upregulation. **c**, A WT *DMD* minigene containing a self-cleaving ribozyme (HHR) cleaves *DMD* pre-mRNA, leading to *UTRN* upregulation. **d**, Splice-switching ASOs disrupting the *DMD* reading frame result in *DMD* mRNA decay and *UTRN* upregulation. RBP, RNA-binding protein. Source Data

Since the identification and analysis of the *mdx* mouse model to study DMD, when utrophin upregulation was observed and shown to compensate for the loss of dystrophin^12^, upregulation of utrophin has been one of the main strategies to treat this disease^46,47^. Nevertheless, the mechanisms underlying utrophin upregulation are poorly understood, and thought to be caused by the loss of dystrophin protein. By using four different approaches, we provide evidence that *DMD* mRNA/pre-mRNA decay is sufficient to upregulate *UTRN*, and also provide evidence that NMD is necessary for *UTRN* upregulation in *DMD*^*PTC/+*^ cells (Extended Data Fig. 10). TA as a mode of genetic compensation was first identified in zebrafish (*Danio rerio*) when investigating differences between mutation-induced phenotypes and antisense (morpholino)-induced phenotypes^6^. Further investigations revealed the presence of TA in *C. elegans*^19^, and identified mutant mRNA decay as playing a key role in triggering TA in zebrafish^8^ and *C. elegans*^19^ as well as in mouse cells in culture^8^. Nevertheless, TA has not been reported in humans thus far. Here we present an example of TA in humans and its potential role in a hereditary disease. Our data show that myotubes from a DMD patient with an E52 deletion display *UTRN* upregulation. Skipping of E51 in these myotubes using an ASO similar to eteplirsen reduced *UTRN* upregulation. For these DMD patients, using *DMD* and/or *UTRN* minigenes with self-cleaving ribozymes might lead to a level of *UTRN* upregulation that could synergize with the benefits of the eteplirsen treatment. Of course, most, if not all, DMD patients might benefit from these self-cleaving ribozyme-containing minigenes. Missense mutations are reportedly more prevalent than frameshift or nonsense mutations, which often lead to mRNA decay, in many human genetic diseases^48–50^. In some of these diseases (for example, sickle cell disease, Marfan syndrome, and hypertrophic cardiomyopathy caused by mutations in *MYH7*), it has been reported that missense mutations have more detrimental effects than nonsense mutations^48–50^. Functional compensation through TA could explain why nonsense mutations are less frequently reported than missense mutations, as they may cause a milder phenotype. ASOs have been approved by the FDA for the treatment of multiple diseases; here, we show a new application for splice-switching ASOs to trigger mutant mRNA decay, and thereby induce functional compensation through TA. Furthermore, minigenes with self-cleaving ribozymes provide another approach to induce TA. This method could be used in patients with missense mutations as well as in those with frameshift or nonsense mutations as increased RNA degradation can further upregulate compensating genes, in line with our previous data^8^. ASOs could similarly be effective in some patients with frameshift or nonsense mutations. We took advantage of *DMD* E37 alternative splicing to create an inducible *DMD* mutant mRNA decay model. This inducible splicing-dependent system constitutes a new tool to induce endogenous mRNA decay. In the last few years, it has been shown that alternative splicing is not an exception but rather more of a rule in multicellular organisms, occurring in roughly 95% of human genes^51^, and genome-wide analyses have revealed that around 20% of a cell’s alternative splicing events are elongation-sensitive^51^. Therefore, this inducible splicing-dependent system holds promise to trigger mRNA decay across a wide range of genes and observing changes over time. Furthermore, in the 2019 Ensembl database of the human genome, about 15,000 alternative splicing variants are annotated as NMD-sensitive isoforms^51^. Inducing the inclusion of elongation-sensitive exons bearing PTCs (poison exons), or inducing PTCs by splice-switching ASOs, also emerge as promising approaches to trigger mRNA decay, without the need to modify the genome. Another potentially promising approach to trigger TA is to use the recently discovered strategy named the proximity-induced nucleic acid degrader in which a small molecule degrades RNA in a targeted manner by being in close proximity to its target^52^. It will also be interesting to test whether RNA interference and/or yet other forms of RNA degradation, can trigger TA or a TA-like response. Altogether, these findings highlight the importance of TA as a mechanism underlying genetic robustness in the human population and its relevance to hereditary diseases, helping in the design of new therapeutic approaches that take advantage of, or do not interfere with, TA-triggered functional compensation.

## Methods

### Dystrophin expression plasmid

The plasmid p37-2iDMD-LR containing the coding sequence for the WT *DMD* gene was a gift from M. Calos (Addgene no. 88892)^53^. *DMD* exons 29 to 34 from p37-2iDMD-LR as well as intron 31 from HEK293T WT genomic DNA were cloned at the 3′ end of the Kozak sequence of the pSBbi-GP plasmid using a Gibson assembly cloning kit (New England Biolabs), resulting in the *DMD*^*WT*^ minigene. A PTC at amino acid position 1421 (E1421X) was introduced in the *DMD*^*WT*^ minigene through site directed mutagenesis to generate the *DMD*^*PTC*^ minigene. *DMD* exons 9 to 11 including intron 10, or exons 34 to 36 including intron 35, from HEK293T WT genomic DNA or complementary DNA (cDNA) were cloned at the 3′ end of the Kozak sequence of the pSBbi-GP plasmid using a HiFi cloning kit (New England Biolabs). The PTCs at amino acid position 338 (E338X) or 1624 (Q1624X) were introduced in the *DMD*^*WT*^ minigenes through site directed mutagenesis to generate the *DMD*^*PTC*^ minigenes. The pSBbi-GP plasmid was a gift from E. Kowarz (Addgene plasmid no. 60511)^54^. The active and inactive versions of the T3H38-HHR ribozyme were inserted in the *DMD*^*WT*^ minigene consisting of exons 29 to 34 and intron 31 by In Vivo Assembly^55^.

### Cell culture and pharmacological treatments

HEK293T cells (DSMZ) and HeLa cells (Enzo Life Sciences) were grown in Dulbecco’s modified Eagle’s medium (DMEM) containing 4.5 g l^−1^ of glucose (Gibco), 10% fetal bovine serum (Sigma) and 1% penicillin streptomycin (Gibco) at 37 °C. HAP1 cells (Horizon Discovery) were grown in Iscove’s modified Dulbecco’s medium containing 10% fetal bovine serum and 1% penicillin streptomycin at 37 °C. Cells were plated at a density of 50,000 cells or 400,000 cells per well in six-well plates 24 h before small-interfering RNA (siRNA) or plasmid transfection, respectively. siRNA (20 nM final concentration), plasmid (1 µg) transfections were performed 24 h after cells were plated using Lipofectamine 3000 (Thermo Fisher Scientific) according to the manufacturer’s instructions. After 24 h, cells were treated with 1 µM TSA (Sigma, T8552), 3 µM CPT (Sigma, C9911) or vehicle for the indicated time and collected for downstream procedures. It should be noted that Trischostatin A and CPT have been reported to have broad effects^56,57^ and that CPT decreases overall transcription^57^ (Supplementary Fig. 2). siRNAs used are listed in Supplementary Table 1. To block translation, WT and *DMD*^*PTC/+*^ cells were treated with 100 µg of cycloheximide (Sigma) or DMSO for the indicated times. For minigene transfections, cells were selected using puromycin (4 µg ml^−1^ for HEK293T cells and 1 µg ml^−1^ for HAP1 and HeLa cells). All cell lines tested negative for mycoplasma contamination.

### Myoblast culture and differentiation

The biopsies used for generating the myoblast lines were supplied by MyoBank, the tissue bank associated with the Institut de Myologie in Paris and affiliated with EuroBioBank. MyoBank is authorized by the French Ministry of Higher Education, Research and Innovation to distribute human samples for research purposes (authorization code AC-2019-3502). Human myoblasts were cultured in Skeletal Muscle Cell Growth Medium (Promocell), adjusted to contain 20% fetal bovine serum and 1% penicillin streptomycin, at 37 °C. They were differentiated by replacing the growth medium with Skeletal Muscle Cell Diffentiation Medium (Promocell), supplemented with 1% penicillin streptomycin, and incubation at 37 °C for 4 to 6 days (ref. ^58^). The characterization of the DMD human myoblast lines (including information about the tissue from which they are derived) is included in Supplementary Table 2.

### C2C12 cell culture and differentiation

C2C12 cells (American Type Culture Collection) were cultured in DMEM containing 4.5 g l^−1^ of glucose, 10% fetal bovine serum and 1% penicillin streptomycin at 37 °C. C2C12 cells were differentiated by replacing the growth medium with DMEM containing 4.5 g l^−1^ of glucose and 2% horse serum and incubation at 37 °C for 3 days.

### Generation of *DMD*^*PTC/+*^ HEK293T cells

HEK293T cells were transfected with the single-guide RNA (5′-GAATGACATACGCCCAAAGG-3′) cloned into the pSpCas9(BB)-2A-Puro plasmid, a gift from F. Zhang (Addgene no. 62988)^59^. HEK293T cells were seeded at a density of 600,000 cells per well in a six-well plate 24 h before plasmid transfection. Plasmid (2.5 µg) transfections were performed using Lipofectamine 3000 according to the manufacturer’s instructions. Cells were incubated for 24 h after transfection, then the transfected cells were selected with medium containing puromycin (4 μg ml^−1^) (Gibco) for 1 week. Puromycin resistant cells were diluted in 10-cm dishes and incubated in fresh medium until single clones formed colonies. Colonies were transferred to a 96-well plate and genomic DNA was isolated from each colony for genotyping with the primer sequences listed in Supplementary Table 3.

### ASO and minigene transfections in skeletal muscle cells

All ASOs used in this study are 18mers uniformly modified with 2′-*O*-MOE ribose, PS linkages and 5′-methylcytosine (sequences in Supplementary Table 4). They were obtained from Integrated DNA Technologies. Human myoblasts were seeded at a density of 100.000 cells per well in a 24-well plate 24 h before ASO or minigene transfection. The myoblasts were transfected with 2.5 or 5 μM ASOs or 1.2 µg of minigene using Lipofectamine 3000 according to the manufacturer’s instructions. For the ASO transfections, the growth medium was replaced with fresh differentiation medium without ASOs 4 h post-transfection and cells were harvested 5 days after transfection. For the minigene transfections, the growth medium was replaced with fresh growth medium without the minigene 4 h post-transfection; fresh differentiation medium was added 24 h later, and cells were harvested 5 days after the differentiation medium was added. In the case of the i51-5ss ASO, where we observed an extra isoform besides the full-length and the E51-skipped isoform, we sequenced the band and found that it was generated by the use of a cryptic splice donor within E51, activated by blocking the splice site at the 5′ end of intron 51. Mouse C2C12 cells were seeded at a density of 60,000 cells per well in a 12-well plate and transfected with 5 μM ASOs using Lipofectamine 3000, according to the manufacturer’s instructions. Then 24 h after transfection, the growth medium was replaced with fresh differentiation medium without ASOs, and cells were harvested 3 days post-transfection. For the C2C12 cells, the differentiation medium was replaced daily until collection.

### RNA extraction and RT–(q)PCR

Cells were collected with 1 ml of Trizol (Invitrogen). Total RNA was isolated according to the manufacturer’s instructions. One microgram of total RNA was reverse transcribed using Superscript III (Thermo Fisher Scientific) reverse transcriptase and oligo-dT primers. The resulting cDNA was amplified using Gotaq (Promega) and *DMD* primers surrounding E6, E22, E37 or E52. After amplification, products were loaded in a 2% agarose gel and stained with SYBR Safe (Invitrogen) for visualization. Primer sequences and product sizes are listed in Supplementary Table 3. Non-consecutive lanes are denoted by a vertical white line of separation in Fig. 1a and Extended Data Figs. 2a, 2d and 9a. For quantitative PCR (qPCR), 1 μg of total RNA was reverse transcribed using a MAXIMA cDNA Synthesis Kit (ThermoFisher Scientific, K1671). qPCR reactions were prepared using DyNAmo ColorFlash SYBR Green PCR mix (Thermo Fisher Scientific, F-416). Primer sequences and products sizes are listed in Supplementary Table 3. A standard program was run on QuantStudio 7 Pro Real-Time PCR System (ThermoFisher Scientific, A43185) and data analysis was performed using Design & Analysis Software v.2.7.0 from ThermoFisher Scientific.

### Western blots

Cells were lysed in RIPA buffer (Sigma). Protein samples were separated by 3–8% Tris-Acetate Protein Gel or 4–20% precast polyacrylamide gel and electroblotted onto PVDF membranes (Bio-Rad). The blots were probed with 1:5,000 anti-Dystrophin (Proteintech, 68120-1-Ig), 1:2,000 anti-Utrophin (Proteintech, 29133-1-AP), 1:2,000 anti-H3K9ac (Proteintech, 29133-1-AP) or 1:2,000 anti-tubulin (Sigma, T6557) antibodies.

### RNA-seq data acquisition and processing

PolyA-enriched RNA-seq was performed for *DMD*^*PTC*/+^ and WT HEK293T cells treated with 1 µM TSA for 24 h. RNA-seq data from myotubes differentiated from iPS cells derived from DMD patients, carrying deletions of exons 45 (DMD1) or 51 (DMD2), both of which lead to PTCs, were obtained from PRJNA1095368 (ref. ^41^). Reads were quality-trimmed using Skewer. The processed reads were aligned to the GRCh38/Gencode v46 genome using STAR, and transcript abundance was estimated using HT-Seq, followed by DESeq2 for differential expression analysis in patient myotubes or IsoDE2 for HEK293T cells. When both experiments were analysed together, batch effects were removed using RUVSeq^60^. DMD1 cells were excluded from the final intersection with our experimental data due to the lack of *DMD* mRNA reduction, suggesting the absence of NMD when compared with control (that is, WT) iPS cells (Extended Data Fig. 6a).

### Statistical analysis

GraphPad Prism v.9 was used for statistical analysis. Two-tailed Student’s *t*-tests were used to compare two pairs of conditions. Unless otherwise stated in the figure legends, we analysed three biologically independent samples with a significance level of *P* < 0.05.

### Reporting summary

Further information on research design is available in the Nature Portfolio Reporting Summary linked to this article.

## Online content

Any methods, additional references, Nature Portfolio reporting summaries, source data, extended data, supplementary information, acknowledgements, peer review information; details of author contributions and competing interests; and statements of data and code availability are available at 10.1038/s41586-024-08539-x.

## Supplementary information

Supplementary Information Supplementary Figs. 1 and 2 and Tables 1–6.

Reporting Summary

## Source data

Source Data Figs. 1–5 and Extended Data Figs. 1–9

## Data Availability

The public dataset generated for this paper, PRJNA1187808, contains RNA-seq data from *DMD*^*PTC/+*^ and WT HEK293T cells treated with TSA. Source data are provided with this paper.

## References

[1] Blake, D. J., Weir, A., Newey, S. E. & Davies, K. E. Function and genetics of dystrophin and dystrophin-related proteins in muscle. *Physiol. Rev.* **82**, 291–329 (2002). 11917091 10.1152/physrev.00028.2001

[2] Duan, D., Goemans, N., Takeda, S., Mercuri, E. & Aartsma-Ru, A. Duchenne muscular dystrophy. *Nat. Rev. Dis. Primers.* **7**, 13 (2021). 33602943 10.1038/s41572-021-00248-3 PMC10557455

[3] Helliwell, T. R., Man, N. T., Morris, G. E. & Davies, K. E. The dystrophin-related protein, utrophin, is expressed on the sarcolemma of regenerating human skeletal-muscle fibers in dystrophies and inflammatory myopathies. *Neuromuscul. Disord.* **2**, 177–184 (1992). 1483043 10.1016/0960-8966(92)90004-p

[4] Anthony, K. et al. Biochemical characterization of patients with in-frame or out-of-frame DMD deletions pertinent to exon 44 or 45 skipping. *JAMA Neurol.* **71**, 32–40 (2014). 24217213 10.1001/jamaneurol.2013.4908

[5] Guiraud, S. & Davies, K. Utrophin correlates with disease severity in Duchenne muscular dystrophy. *Med.* **4**, 220–222 (2023). 37060897 10.1016/j.medj.2023.03.005

[6] Rossi, A. et al. Genetic compensation induced by deleterious mutations but not gene knockdowns. *Nature* **524**, 230–233 (2015). 26168398 10.1038/nature14580

[7] El-Brolosy, M. A. & Stainier, D. Y. R. Genetic compensation: a phenomenon in search of mechanisms. *PLoS Genet.* **13**, e1006780 (2017). 28704371 10.1371/journal.pgen.1006780 PMC5509088

[8] El-Brolosy, M. A. et al. Genetic compensation triggered by mutant mRNA degradation. *Nature* **568**, 193–197 (2019). 30944477 10.1038/s41586-019-1064-z PMC6707827

[9] Mendell, J. R. et al. Eteplirsen for the treatment of Duchenne muscular dystrophy. *Ann. Neurol.* **74**, 637–647 (2013). 23907995 10.1002/ana.23982

[10] Bushby, K. et al. Ataluren treatment of patients with nonsense mutation dystrophinopathy. *Muscle Nerve* **50**, 477–487 (2014). 25042182 10.1002/mus.24332 PMC4241581

[11] Duan, D., Luo, J. & Zhang, Y. AAV-mediated micro-dystrophin gene therapy in dystrophin-deficient mice. *Mol. Ther.* **26**, 2975–2986 (2018).

[12] Deconinck, A. E. et al. Utrophin-dystrophin-deficient mice as a model for Duchenne muscular dystrophy. *Cell.* **90**, 717–727 (1997). 9288751 10.1016/s0092-8674(00)80532-2

[13] Law, D. J., Allen, D. L. & Tidball, J. G. Talin, vinculin and DRP (utrophin) concentrations are increased at mdx myotendinous junctions following onset of necrosis. *J. Cell Sci.* **107**, 1477–1483 (1994). 7962191 10.1242/jcs.107.6.1477

[14] Georgieva, A. M. et al. Inactivation of Sirt6 ameliorates muscular dystrophy in *mdx* mice by releasing suppression of utrophin expression. *Nat. Commun.* **13**, 4184 (2022). 35859073 10.1038/s41467-022-31798-z PMC9300598

[15] Janghra, N. et al. Correlation of utrophin levels with the dystrophin protein complex and muscle fibre regeneration in Duchenne and Becker muscular dystrophy muscle biopsies. *PLoS ONE* **11**, e0150818 (2016). 26974331 10.1371/journal.pone.0150818 PMC4790853

[16] Kleopa, K. A., Drousiotou, A., Mavrikiou, E., Ormiston, A. & Kyriakides, T. Naturally occurring utrophin correlates with disease severity in Duchenne muscular dystrophy. *Hum. Mol. Genet.* **15**, 1623–1628 (2006). 16595608 10.1093/hmg/ddl083

[17] Masubuchi, N., Shidoh, Y., Kondo, S., Takatoh, J. & Hanaoka, K. Subcellular localization of dystrophin isoforms in cardiomyocytes and phenotypic analysis of dystrophin-deficient mice reveal cardiac myopathy is predominantly caused by a deficiency in full-length dystrophin. *Exp Anim.* **62**, 211–217 (2013). 23903056 10.1538/expanim.62.211 PMC4160940

[18] Ma, Z. et al. PTC-bearing mRNA elicits a genetic compensation response via Upf3a and COMPASS components. *Nature* **568**, 259–263 (2019). 30944473 10.1038/s41586-019-1057-y

[19] Serobyan, V. et al. Transcriptional adaptation in *Caenorhabditis elegans*. *eLife* **9**, e50014 (2020). 31951195 10.7554/eLife.50014 PMC6968918

[20] Kontarakis, Z. & Stainier, D. Y. R. Genetics in light of transcriptional adaptation. *Trends Genet.* **36**, 926–935 (2020). 32928563 10.1016/j.tig.2020.08.008 PMC7612536

[21] Sztal, T. E. & Stainier, D. Y. R. Transcriptional adaptation: a mechanism underlying genetic robustness. *Development* **147**, dev186452 (2020). 32816903 10.1242/dev.186452

[22] Jakutis, G. & Stainier, D. Y. R. Genotype–phenotype relationships in the context of transcriptional adaptation and genetic robustness. *Annu. Rev. Genet.* **55**, 71–91 (2021). 34314597 10.1146/annurev-genet-071719-020342

[23] Jiang, Z. et al. Parental mutations influence wild-type offspring via transcriptional adaptation. *Sci. Adv.* **8**, eabj2029 (2022). 36427314 10.1126/sciadv.abj2029 PMC9699682

[24] Fernandez-Abascal, J., Wang, L., Graziano, B., Johnson, C. K. & Bianchi, L. Exon dependent transcriptional adaptation by exon-junction complex proteins Y14/RNP-4 and MAGOH/MAG-1 in *Caenorhabditis elegans*. *PLoS Genet.* **18**, e1010488 (2022). 36315586 10.1371/journal.pgen.1010488 PMC9648848

[25] Welker, J. M., Serobyan, V., Esfahani, E. Z. & Stainier, D. Y. R. Partial sequence identity in a 25-nucleotide long element is sufficient for transcriptional adaptation in the *Caenorhabditis elegans* act-5/act-3 model. *PLoS Genet.* **19**, e1010806 (2023). 37384903 10.1371/journal.pgen.1010806 PMC10310345

[26] Tuffery-Giraud, S. et al. Genotype-phenotype analysis in 2,405 patients with a dystrophinopathy using the UMD-DMD database: a model of nationwide knowledgebase. *Hum. Mutat.* **30**, 934–945 (2009). 19367636 10.1002/humu.20976

[27] Juan-Mateu, J. et al. Interplay between DMD point mutations and splicing signals in dystrophinopathy phenotypes. *PLoS ONE* **8**, e59916 (2013). 23536893 10.1371/journal.pone.0059916 PMC3607557

[28] Flanigan, K. M. et al. Nonsense mutation-associated Becker muscular dystrophy: interplay between exon definition and splicing regulatory elements within the DMD gene. *Hum. Mutat.* **32**, 299–308 (2011). 21972111 10.1002/humu.21426 PMC3724403

[29] Boireau, S. et al. The transcriptional cycle of HIV-1 in real-time and live cells. *J. Cell Biol.* **179**, 291–304 (2007). 17954611 10.1083/jcb.200706018 PMC2064765

[30] Darzacq, X. et al. In vivo dynamics of RNA polymerase II transcription. *Nat. Struct. Mol. Biol.* **14**, 796–806 (2007). 17676063 10.1038/nsmb1280 PMC4942130

[31] Dujardin, G. et al. How slow RNA polymerase II elongation favors alternative exon skipping. *Mol. Cell* **54**, 683–690 (2014). 24793692 10.1016/j.molcel.2014.03.044

[32] Listerman, I., Sapra, A. K. & Neugebauer, K. M. Cotranscriptional coupling of splicing factor recruitment and precursor messenger RNA splicing in mammalian cells. *Nat. Struct. Mol. Biol.* **13**, 815–822 (2006). 16921380 10.1038/nsmb1135

[33] Marasco, L. E. et al. Counteracting chromatin effects of a splicing-correcting antisense oligonucleotide improves its therapeutic efficacy in spinal muscular atrophy. *Cell* **185**, 2057–2070 (2022). 35688133 10.1016/j.cell.2022.04.031 PMC9555286

[34] Lalonde, S. et al. Frameshift indels introduced by genome editing can lead to in-frame exon skipping. *PLoS ONE* **12**, e0178700 (2017). 28570605 10.1371/journal.pone.0178700 PMC5453576

[35] Anderson, J. L. et al. mRNA processing in mutant zebrafish lines generated by chemical and CRISPR-mediated mutagenesis produces unexpected transcripts that escape nonsense-mediated decay. *PLoS Genet.* **13**, e1007105 (2017). 29161261 10.1371/journal.pgen.1007105 PMC5716581

[36] Cartegni, L., Wang, J., Zhu, Z., Zhang, M. Q. & Krainer, A. R. ESEfinder: a web resource to identify exonic splicing enhancers. *Nucleic Acids Res.* **31**, 3568–3571 (2003). 12824367 10.1093/nar/gkg616 PMC169022

[37] Maquat, L. E. Nonsense-mediated mRNA decay in mammals. *J. Cell Sci.* **118**, 1773–1776 (2005). 15860725 10.1242/jcs.01701

[38] Monaghan, L., Longman, D. & Cáceres, J. F. Translation-coupled mRNA quality control mechanisms. *EMBO J.* **42**, e114378 (2023). 37605642 10.15252/embj.2023114378 PMC10548175

[39] McCarthy, J. J., Esser, K. A. & Andrade, F. H. MicroRNA-206 is overexpressed in the diaphragm but not the hindlimb muscle of mdx mouse. *Am. J. Physiol. Cell Physiol.* **293**, C451–C457 (2007). 17459947 10.1152/ajpcell.00077.2007

[40] Verhaart, I. E. et al. The dynamics of compound, transcript, and protein effects after treatment with 2OMePS antisense oligonucleotides in *mdx* mice. *Mol. Ther. Nucleic Acids* **3**, e148 (2014). 24549299 10.1038/mtna.2014.1 PMC3950770

[41] Dhoke, N. R. et al. A novel CRISPR–Cas9 strategy to target DYSTROPHIN mutations downstream of exon 44 in patient-specific DMD iPSCs. *Cells* **13**, 972 (2024). 38891104 10.3390/cells13110972 PMC11171783

[42] Zhong, G. et al. A reversible RNA on-switch that controls gene expression of AAV-delivered therapeutics in vivo. *Nat. Biotechnol.* **38**, 169–175 (2020). 31873216 10.1038/s41587-019-0357-y PMC7008088

[43] Doherty, E. A. & Doudna, J. A. Ribozyme structures and mechanisms. *Annu. Rev. Biochem.* **69**, 597–615 (2000). 10966470 10.1146/annurev.biochem.69.1.597

[44] Arechavala-Gomeza, V. et al. Comparative analysis of antisense oligonucleotide sequences for targeted skipping of exon 51 during dystrophin pre-mRNA splicing in human muscle. *Hum. Gene Ther.* **18**, 798–810 (2007). 17767400 10.1089/hum.2006.061

[45] Galli, F. et al. Cell-mediated exon skipping normalizes dystrophin expression and muscle function in a new mouse model of Duchenne muscular dystrophy. *EMBO Mol. Med*. 10.1038/s44321-024-00031-3 (2024). 10.1038/s44321-024-00134-x PMC11473694 39242974

[46] Tinsley, J. M. et al. Amelioration of the dystrophic phenotype of mdx mice using a truncated utrophin transgene. *Nature* **384**, 349–353 (1996). 8934518 10.1038/384349a0

[47] Sengupta, K., Loro, E. & Khurana, T. S. PMO-based let-7c site blocking oligonucleotide (SBO) mediated utrophin upregulation in mdx mice, a therapeutic approach for Duchenne muscular dystrophy (DMD). *Sci. Rep.* **10**, 21492 (2020). 33298994 10.1038/s41598-020-76338-1 PMC7726560

[48] Carlice-Dos-Reis, T. et al. Investigation of mutations in the HBB gene using the 1,000 genomes database. *PLoS ONE* **12**, e0174637 (2017). 28379995 10.1371/journal.pone.0174637 PMC5381778

[49] Kelly, M. A. et al. Adaptation and validation of the ACMG/AMP variant classification framework for MYH7-associated inherited cardiomyopathies: recommendations by ClinGen’s Inherited Cardiomyopathy Expert Panel. *Genet. Med.* **20**, 351–359 (2018). 29300372 10.1038/gim.2017.218 PMC5876064

[50] Dietz, H. C. et al. Four novel FBN1 mutations: significance for mutant transcript level and EGF-like domain calcium binding in the pathogenesis of Marfan syndrome. *Genomics* **17**, 468–475 (1993). 8406497 10.1006/geno.1993.1349

[51] Marasco, L. E. & Kornblihtt, A. R. The physiology of alternative splicing. *Nat. Rev. Mol. Cell Biol.* **24**, 242–254 (2022). 36229538 10.1038/s41580-022-00545-z

[52] Mikutis, S. et al. Proximity-induced nucleic acid degrader (PINAD) approach to targeted RNA degradation using small molecules. *ACS Cent. Sci.* **9**, 892–904 (2023). 37252343 10.1021/acscentsci.3c00015 PMC10214512

[53] Farruggio, A. P. et al. Genomic integration of the full-length dystrophin coding sequence in Duchenne muscular dystrophy induced pluripotent stem cells. *Biotechnol. J.*10.1002/biot.201600477 (2017). 10.1002/biot.201600477 28139886

[54] Kowarz, E. et al. Optimized Sleeping Beauty transposons rapidly generate stable transgenic cell lines. *Biotechnol. J.* **10**, 647–653 (2015). 25650551 10.1002/biot.201400821

[55] García-Nafría, J., Watson, J. F. & Greger, I. H. IVA cloning: a single-tube universal cloning system exploiting bacterial in vivo assembly. *Sci. Rep.* **6**, 27459 (2016). 27264908 10.1038/srep27459 PMC4893743

[56] Bouyahya, A. et al. Pharmacological properties of trichostatin A, focusing on the anticancer potential: a comprehensive review. *Pharmaceuticals* **15**, 1235 (2022). 36297347 10.3390/ph15101235 PMC9612318

[57] Veloso, A. et al. Genome-wide transcriptional effects of the anti-cancer agent camptothecin. *PLoS ONE* **8**, e78190 (2013). 24194914 10.1371/journal.pone.0078190 PMC3806802

[58] Mamchaoui, K. et al. Immortalized pathological human myoblasts: towards a universal tool for the study of neuromuscular disorders. *Skelet. Muscle* **1**, 34 (2011). 22040608 10.1186/2044-5040-1-34 PMC3235972

[59] Ran, F. A. et al. Genome engineering using the CRISPR-Cas9 system. *Nat. Protocols* **8**, 2281–2308 (2013). 24157548 10.1038/nprot.2013.143 PMC3969860

[60] Mandric, I. et al. Fast bootstrapping-based estimation of confidence intervals of expression levels and differential expression from RNA-seq data. *Bioinformatics* **33**, 3302–3304 (2017). 28605502 10.1093/bioinformatics/btx365

