# Supplementary material for: Transcriptional adaptation upregulates utrophin in Duchenne muscular dystrophy
Source: Nature. 2025 Feb 12;639(8054):493–502. doi: 10.1038/s41586-024-08539-x (PMC11903304; doi:10.1038/s41586-024-08539-x)
Supplement: Supplementary file 1 — Supplementary Figs. 1 and 2 and Tables 1–6. [file 41586_2024_8539_MOESM1_ESM.pdf]

nature portfolio

https://doi.org/10.1038/s41586-024-08539-x

Supplementary information

Transcriptional adaptation upregulates utrophin in Duchenne muscular dystrophy

In the format provided by the authors and unedited

Supplementary Information for:

Transcriptional adaptation upregulates utrophin in Duchenne muscular dystrophy

Lara Falcucci, Christopher M. Dooley, Justin Martinez, Douglas Adamoski, Thomas Juan, Angelina M. Georgieva, Kamel Mamchaoui, Cansu Cirzi, Didier Y. R. Stainier

**Supplementary Figures**	**2**
Supplementary Fig. 1	2
Supplementary Fig. 2	5
**Supplementary Tables**	**6**
Supplementary Table 1	6
Supplementary Table 2	6
Supplementary Table 3	6
Supplementary Table 4	7
Supplementary Table 5	7
Supplementary Table 6	9

Supplementary Fig. 1

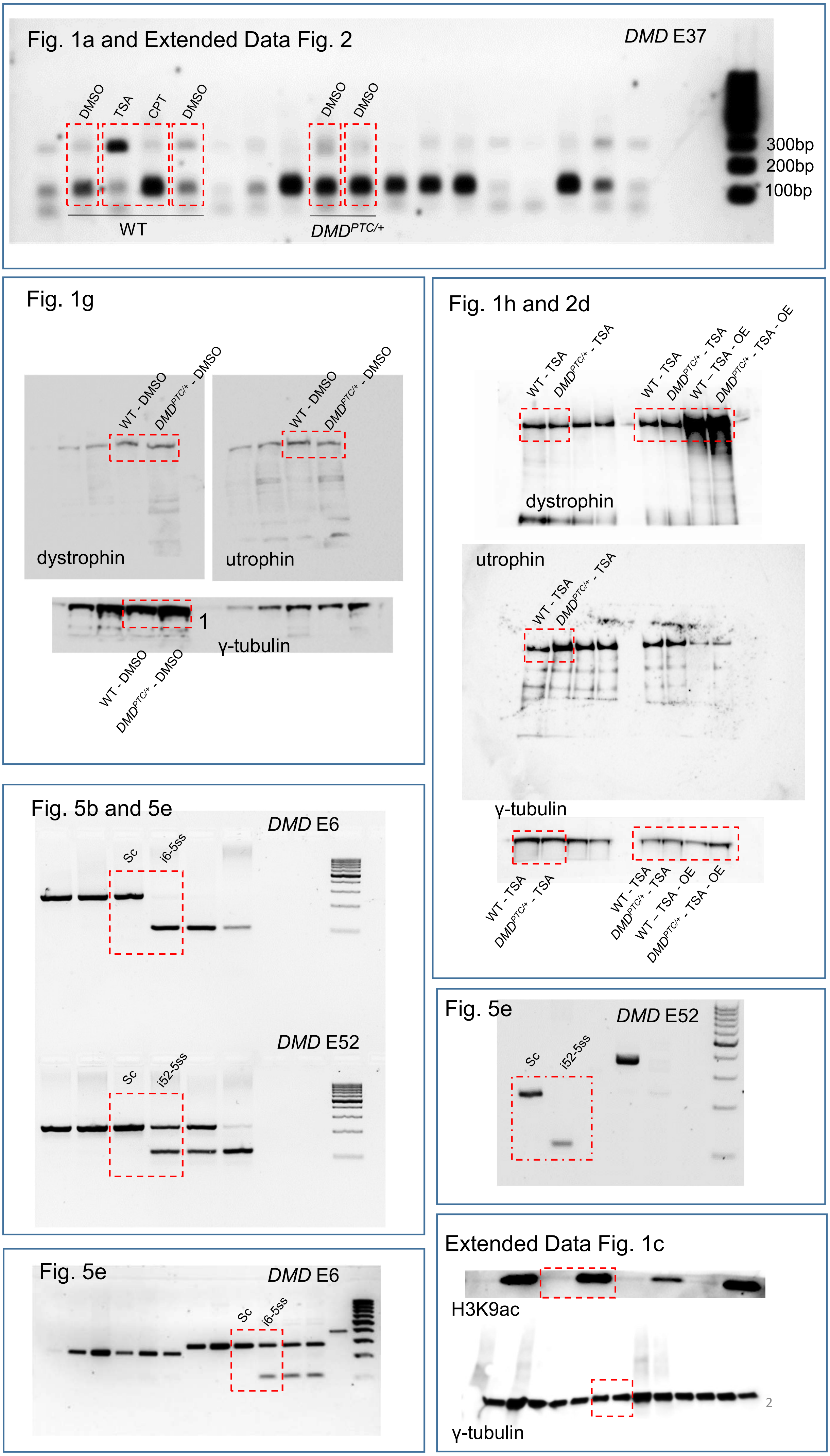

Supplementary Fig. 1

Fig. 4c and Extended Data Fig. 9a

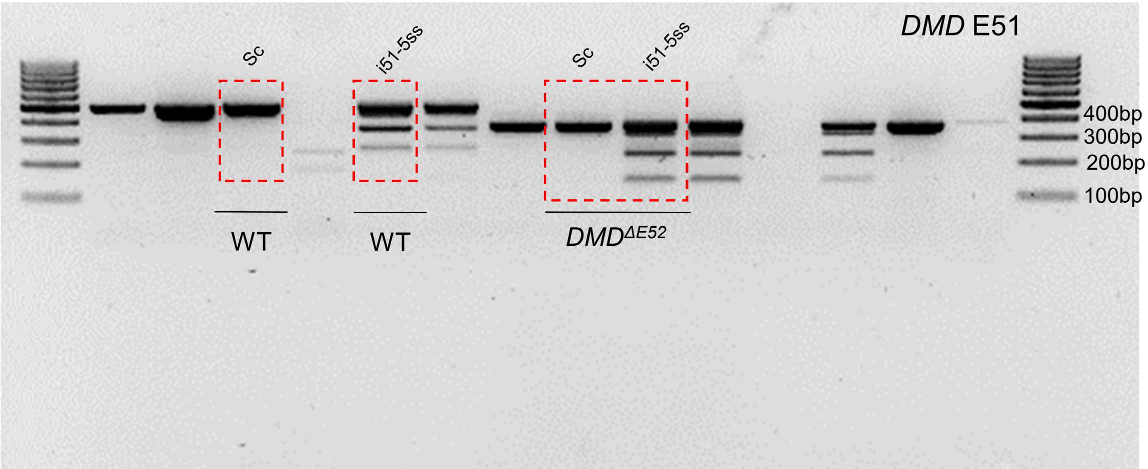

Fig. 4e

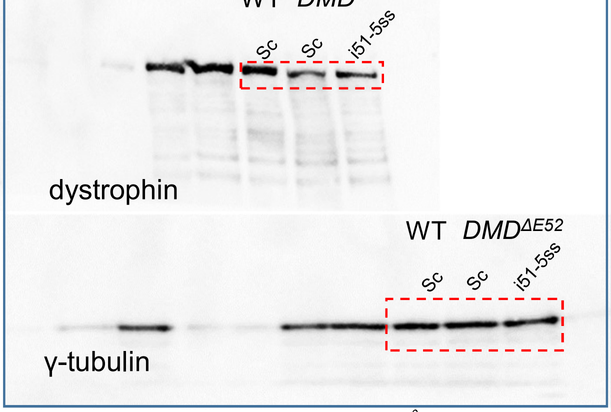

Fig. 5h

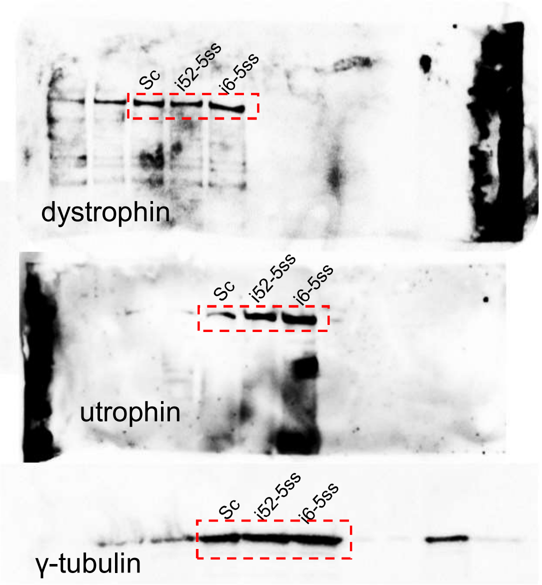

Fig. 4f

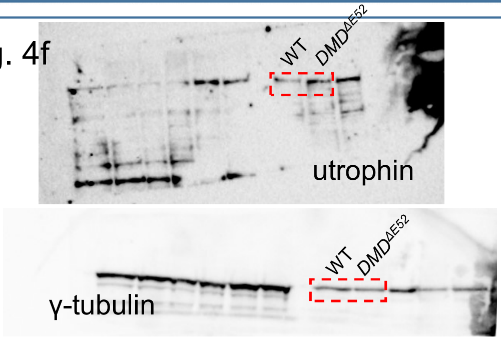

Fig. 4f

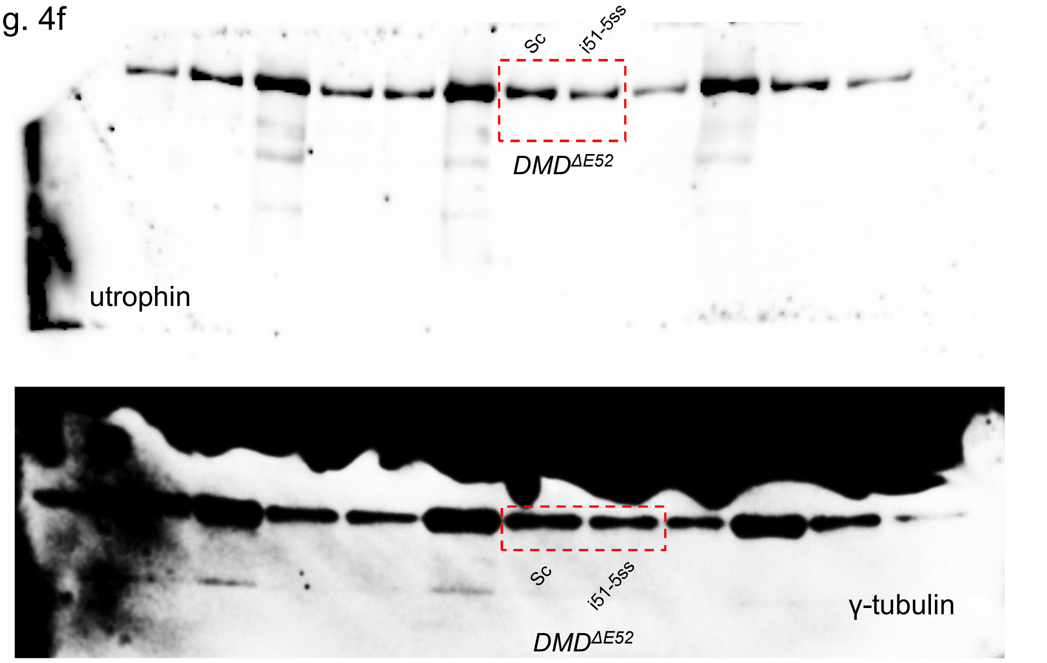

Supplementary Fig. 1

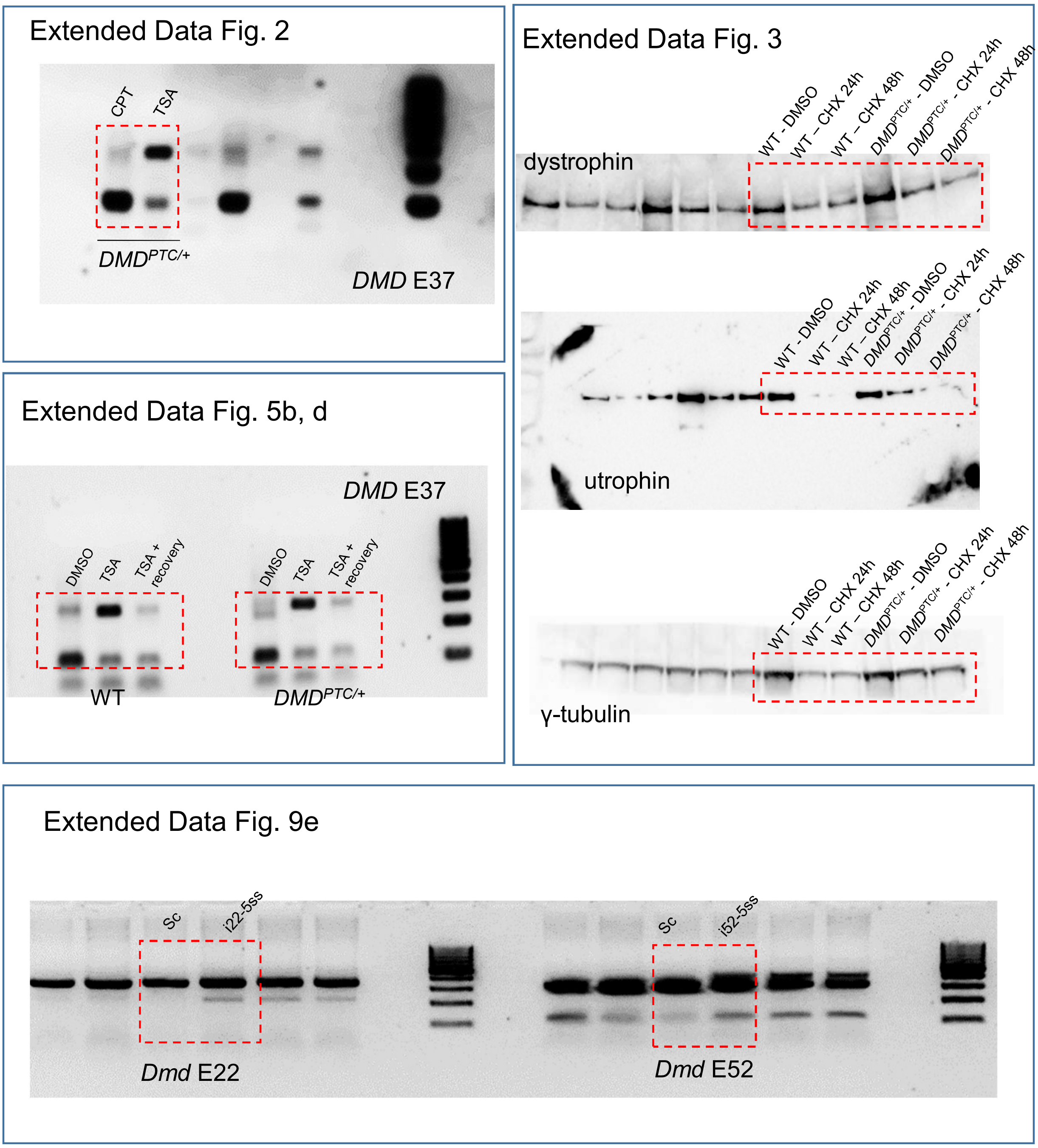

Figure S1. **Expanded view of blots and gels images.**
Expanded (uncropped) view of gel and blot images used in Main Figures and Extended Data Figures with molecular weight markers.

Supplementary Fig. 2

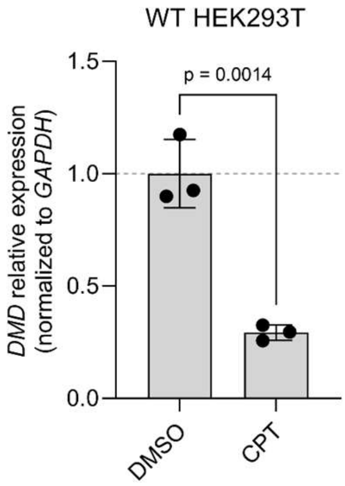

Figure S2. **CPT decreases *DMD* transcription.**
qPCR analysis of *DMD* mRNA levels in WT HEK293T cells treated with DMSO or 3 µm CPT for 6 h. Data are normalized to WT treated with DMSO and are mean ± s.d.; a two-tailed Student's *t*-test was used to calculate p values (n = 3 biologically independent samples).

Supplementary Table 1. List of siRNAs used.

ON-TARGET plus Human SMG6	CCAGUGAUACAGCGAAUUA	Dharmacon
ON-TARGET plus Human UPF1	CAGCGGAUCGUGUGAAGAA	Dharmacon
ON-TARGET plus Non-targeting pool	UGGUUUACAUGUCGACUAA,<br>UGGUUUACAUGUUGUGUGA,<br>UGGUUUACAUGUUUUCUGA,<br>UGGUUUACAUGUUUUCCUA	Dharmacon

Supplementary Table 2. Characterization of human myoblasts

Human myoblasts	Origin	Genomic lesion in *DMD*
WT	paravertebral derived from a 16 year old male or from a 13 year old female	-
DMD^{E118X}	paravertebral derived from a 14 year old male	c35456G-T<br>(p.Glu118X)
$DMD^{\Delta E51\text{-}54}$	paravertebral derived from a 13 year old male	ΔE51-54
$DMD^{\Delta E52}$	paravertebral derived from a 16 year old male	ΔE52
DMD^{R2905X}	quadriceps derived from a 11 year old male	c.8713C>T<br>(p.Arg2905X)

Supplementary Table 3. List of primers used.

Primer	Sequence	Product size (nt)
DMD E37_sgRNA	5′– GAATGACATACGCCCAAAGG – 3′	-
F_*DMD* E37_genotyping	5′– ACGCATTTTCATTTTAGTCCTGT – 3′	681
R_*DMD* E37_ genotyping	5′– GGTGACCCCTGAAAGTACGT – 3′	
F_*DMD* E37_RT-PCR	5′– ACCCCAGCAAAAAGAAGACGTGC – 3′	274 (E37^{+})/103 (E37^{-})
R_*DMD* E37_RT-PCR	5′– TTCAGCCTCCAGTGGTTCAAGC – 3′	
F_*DMD* E51_RT-PCR	5′– CTCTGAGTGGAAGGCGGTAA – 3′	$DMD^{\Delta E52}$<br>378 (E51^{+})/145 (E51^{-})<br>WT<br>496 (E51^{+})/263 (E51^{-})
R_*DMD* E51_RT-PCR	5′– CCTCCGGTTCTGAAGGTGTT – 3′	
F_*DMD* E52_RT-PCR	5′– CAGAGGGTGATGGTGGGTG – 3′	231 (E52^{+})/113 (E52^{-})
R_*DMD* E52_RT-PCR	5′– CCTCCGGTTCTGAAGGTGTT – 3′	
F_*DMD* E6_RT-PCR	5′– ACTGACTCTTGGTTTGATTTGGA – 3′	282 (E6^{+})/109 (E6^{-})
R_*DMD* E6_RT-PCR	5′– GCATGTTCCAGTCGTTGTGT – 3′	
F_*DMD* E37_qPCR	5′– ACCCCAGCAAAAAGAAGACGTGC – 3′	231 (E37^{+})/60 – no product (E37^{-})
R_*DMD* E37_qPCR	5′– AACTGCTCCAATTCCTTCAAAGG – 3′	
F_*Dmd* E22_RT-PCR	5′– GGATGCAGACTTTGTGGCC – 3′	372 (E22^{+})/226 (E22^{-})
R_*Dmd* E22_RT-PCR	5′– TTCTTCCAGTGCCCCTCAAT – 3′	
F_*Dmd* E52_RT-PCR	5′– ACTGGCTGTCTCTGCTTGAT – 3′	244 (E52^{+})/126 (E52^{-})
R_*Dmd* E52_RT-PCR	5′– CCTGTTCGGCTTCTTCCTTAG – 3′	
F_*DMD* mRNA myo_qPCR	5′– TGCTCTCATCCATAGTCATAGGCC – 3′	162
R_ *DMD* mRNA myo_qPCR	5′– TCTGGATAGGTGGTATCAACATCT – 3′	
F_*DMD* mRNA_qPCR	5′– CAGATGAGAGAAAGCGAGAGG – 3′	155
R_ *DMD* mRNA_qPCR	5′– CAGGAGATCATCAGCCTGCC – 3′	
F_ *DMD*mini E29-34 _qPCR	5′– GCTGCCCAAAGAGTCCTGTC – 3′	103
R_ *DMD*mini E29-34 _qPCR	5′– GCTGCTCAAAATTGGCTGGTT – 3′	
F_ *DMD*mini E34-36 _qPCR	5′– TCCCGAGCAGAAGAGTGGT – 3′	119
R_ *DMD*mini E34-36 _qPCR	5′– TCATCCAAAAGTGTGTCAGCCT – 3′	
F_ *DMD*mini E9-11 _qPCR	5′– TGGCTTCTTTCTGCTGAGGAC – 3′	250
R_ *DMD*mini E9-11 _qPCR	5′– CTACCCTGAGGCATTCCCATC – 3′	
F_*UTRN* mRNA_qPCR	5′– CGAACAGCAAAAGGTCACAA – 3′	158
R_ *UTRN* mRNA_qPCR	5′– ACAGGCAGGTAACCAAGTCG – 3′	
F_*UTRN* pre-mRNA_qPCR	5′– AGTGGGAAACCACCCATCAA – 3′	141
R_ *UTRN* pre-mRNA_qPCR	5′– ATCAGCAATGACCGGAGACT – 3′	
F_*DMD* pre-mRNA_qPCR	5′– AACTTTGCACAAATTGTGAGTTGT– 3′	172
R_ *DMD* pre-mRNA_qPCR	5′– AGAAGCCCAAAGTGAGGGAA– 3′	
F_*Hprt* mRNA_qPCR	5′– AGGGATTTGAATCACGTTTG – 3′	116
R_ *Hprt* mRNA_qPCR	5′– TTTACTGGCAACATCAACAG – 3′	
F_*Utrn* mRNA_qPCR	5′– GCCATCATCTTGGTGAATGCTCG – 3′	141
R_ *Utrn* mRNA_qPCR	5′– GGATGAAGGGTCCTGACCAATC – 3′	
F_*Dmd* mRNA_qPCR	5′– AGCTCAACCGTCGATTTGCAGC – 3′	125
R_ *Dmd* mRNA_qPCR	5′– TTCAGCCTCCAGTGGTTCAAGC – 3′	
F_*UPF1* mRNA_qPCR	5′– AACGAGCACCAAGGCATTGGCT – 3′	116
R_ *UPF1* mRNA_qPCR	5′– GGCTGCTTTGATAGTGCCTTCG – 3′	
F_*SMG6* mRNA_qPCR	5′– TAGCAGCAGCCGCGAAG – 3′	121
R_ *SMG6* mRNA_qPCR	5′– AATTCCTTCATGTTTTCTCTGCTC – 3′	
F_*GAPDH* mRNA_qPCR	5′– GTCTCCTCTGACTTCAACAGCG – 3′	131
R_ *GAPDH* mRNA_qPCR	5′– ACCACCCTGTTGCTGTAGCCAA – 3′	

Supplementary Table 4. List of antisense oligonucleotides used.

ASO type	ASO name	Sequence
Exon skip	i52-5ss	5′– GCTTGTTAAAAAACTTAC – 3′
Exon skip	i51-5ss	5′– TATCATTTTTTCTCATAC – 3′
Exon skip	i6-5ss	5′– TCTCAGTAATCTTCTTAC – 3′
Exon skip	i22-5ss (mouse)	5′– ATTCAAATGTCCACAGAC – 3′
Exon skip	i52-5ss (mouse)	5′– GCCCATTAAGAGACTTAC – 3′
Scrambled control[59]	Sc	5′– ACTTCGTTCGCCTTAACT – 3′

Supplementary Table 5. List of pooled Ct values for Figures 1 to 5, Extended Data Figures 1 to 3, and Extended Data Figures 7 to 9.

Average Ct values

	DMD E37 mRNA	*DMD* mRNA	*UTRN* mRNA	*UTRN* pre-mRNA	*GAPDH* mRNA
WT					
DMSO	32.513	26.375	22.003	26.205	14.932
CPT	33.285	-	-	-	14.665
TSA 1 μM 24 h	29.262	26.763	23.672	28.582	15.256
DMD$^{PTC/+}$					
DMSO	33.346	26.456	21.529	26.969	14.624
CPT	33.904	-	-	-	14.728
TSA 1 μM 24 h	29.498	27.269	22.672	27.933	15.290
TSA 0.1 μM	32.430	25.781	21.414	-	15.233
TSA + recovery	31.545	26.704	21.026	-	15.180

	DMD mRNA	*UTRN* mRNA	*UTRN* pre-mRNA	*GAPDH* mRNA
WT				
siCTRL	29.032	26.386	31.404	16.838
siUPF1+ siSMG6	29.298	25.787	30.869	16.445
DMD$^{PTC/+}$				
siCTRL	29.481	25.118	30.229	16.746
siUPF1+ siSMG6	28.834	25.596	30.400	16.554

	UTRN mRNA	*UTRN* pre-mRNA	*GAPDH* mRNA
WT			
TSA 1 μM + *DMD* OE	21.749	27.307	15.303
DMD$^{PTC/+}$			
TSA 1 μM + *DMD* OE	21.685	26.934	15.755

	DMD mRNA	*UTRN* mRNA	*GAPDH* mRNA
WT HEK293T			
Empty	27.43465	21.79581	15.88479
DMD WT E338	8.288935	20.39122	14.87878
DMD E338X	8.860208	19.702271	13.9337
Empty	28.10689	20.9433	13.85495
DMD WT E1421	8.254478	21.09524	13.94884
DMD E1421X	9.599149	20.33504	14.06659
Empty	31.1933	21.95069	15.34861
DMD WT E1624	9.917438	22.23017	15.31646
DMD E1624X	12.96904	21.32822	14.83038
T3H38-iHHR	10.99472	24.575	14.07442
T3H38-aHHR	13.36811	24.20037	15.36323
WT myotubes			
Empty	19.8234	21.17312	15.50801
DMD WT E338	15.98911	21.1734	15.6489
DMD E338X	16.68313	12.23856	15.75137
Empty	26.625	25.506	17.345
DMD WT E1421	15.363	24.807	16.867
DMD E1421X	15.765	24.638	17.121
Empty	21.08324	21.34416	15.50801
DMD WT E1624	17.42094	21.31036	15.68029
DMD E1624X	17.79777	21.12173	15.76675
T3H38-iHHR	16.74788	25.34597	15.93629

T3H38-aHHR	18.10594		24.52618	16.52637
Sc	21.365		26.622	17.225
i52-5ss (full skipping)	21.8		25.464	17.332
Sc	23.907		27.055	18.571
i6-5ss (full skipping)	24.657		26.090	18.317
Sc	17.827		21.253	18.507
i52-5ss	17.627		19.771	18.447
Sc	22.604		23.552	16.575
i6-5ss	22.913		22.833	16.779
Sc	19.996		19.255	15.839
i51-5ss	21.865		21.479	15.867
WT HAP1				
Empty	30.40653		20.30734	13.75306
DMD WT E338	15.19167		20.15157	14.07627
DMD E338X	15.99867		20.61295	15.03356
Empty	32.911		23.869	16.004
DMD WT E1421	18.314		25.243	17.464
DMD E1421X	20.956		25.978	18.741
Empty	27.83631		20.19014	14.63824
DMD WT E1624	15.47215		20.38714	14.48652
DMD E1624X	15.75464		20.40697	14.89879
T3H38-iHHR	26.1612		25.64586	20.68826
T3H38-aHHR	26.01146		23.82442	19.59125
WT HeLa				
Empty	32.22221		24.88421	18.11484
DMD WT E338	16.26438		23.68777	16.77796
DMD E338X	17.85865		23.75521	17.11822
Empty	33.42278		24.88421	18.11484
DMD WT E1421	17.68026		24.16947	17.54922
DMD E1421X	18.0477		23.92044	17.24499
Empty	32.03663		24.88421	18.11484
DMD WT E1624	17.16801		23.45686	16.55282
DMD E1624X	17.79623		23.12772	16.33812
T3H38-iHHR	17.37719		24.37304	16.05185
T3H38-aHHR	19.56194		24.69834	16.17
	DMD pre-mRNA		*GAPDH* mRNA	
WT myotubes				
Sc	31.228		15.409	
i51-5ss	29.731		15.054	
	DMD mRNA	*UTRN* mRNA	*UTRN pre-mRNA*	*GAPDH* mRNA
Human myotubes				
WT	21.326	24.006	29.786	18.350
$DMD^{\Delta E52}$	27.493	24.273	30.296	19.924
DMD^{E118X}	22.549	23.381	27.998	18.897
WT	19.910	23.012	29.787	16.778
DMD^{R2905X}	21.266	23.938	27.72	17.586
WT	19.755	22.897	29.786	16.581
$DMD^{\Delta E51\text{-}54}$	20.116	21.933	28.104	16.828
	DMD mRNA		*UTRN* mRNA	*GAPDH* mRNA
$DMD^{\Delta E52}$ myotubes				
T3H38-iHHR	16.247		22.065	15.893
T3H38-aHHR	18.413		21.158	15.910
Sc	23.024		22.419	15.071
i51-5ss	20.057		26.621	14.647
DMD^{R2905X} myotubes				
T3H38-iHHR	15.515		23.867	16.334
T3H38-aHHR	17.864		23.421	16.841
$DMD^{\Delta E51\text{-}54}$ myotubes				
T3H38-iHHR	16.452		24.851	17.109
T3H38-aHHR	18.993		24.084	17.340
	Utrn mRNA		*Hprt* mRNA	
C2C12 skeletal muscle				
Sc	20.525		17.630	
i22-5ss	20.089		17.497	
i52-5ss	20.426		18.001	
	Dmd mRNA		*Hprt* mRNA	
C2C12 skeletal muscle				
Sc	25.587		18.138	
i22-5ss	25.421		18.090	
i52-5ss	25.426		18.072	

Supplementary Table 6. List of pooled Ct values for Extended Data Figures 4 to 5.

Average Ct values					
	DMD E37 mRNA	*DMD* mRNA	*UTRN* mRNA	*UTRN* pre-mRNA	*GAPDH* mRNA
WT					
DMSO	32.513	26.375	22.003	26.205	14.932
TSA 1 µM 24 h	29.262	26.763	23.672	28.582	15.256
TSA 1 µM 16 h	28.783	25.624	22.619	-	15.028
TSA 1 µM 8 h	32.621	25.229	24.076	-	15.927
TSA 1 µM 48 h	31.722	28.495	27.072	-	19.357
TSA 0.5 µM	29.542	26.994	23.066	-	14.747
TSA 0.1 µM	32.430	25.781	21.414	-	15.233
TSA + recovery	31.545	26.704	21.026	-	15.180
DMD$^{PTC/+}$					
DMSO	33.346	26.456	21.529	26.969	14.624
TSA 1 µM 24 h	29.498	27.269	22.672	27.933	15.290
TSA 1 µM 16 h	29.407	26.034	22.221	-	15.259
TSA 1 µM 8 h	32.426	25.687	22.479	-	14.923
TSA 1 µM 48 h	32.419	29.728	28.447	-	20.825
TSA 0.5 µM	29.506	26.978	22.488	-	14.465
TSA 0.1 µM	33.625	26.463	21.090	-	15.078
TSA + recovery	30.771	27.004	20.716	-	15.148

	UPF1 mRNA	*SMG6* mRNA	*GAPDH* mRNA		*UPF 1* mRNA	*SMG6* mRNA	*GAPDH* mRNA
WT				*DMD*$^{PTC/+}$			
siCTRL	23.14	24.159	16.838	siCTRL	24.074	25.109	16.746

58. Kim Y.J., Sivetz N., Layne J., Voss D.M., Yang L., Zhang Q., Krainer A.R. Exon-skipping antisense oligonucleotides for cystic fibrosis therapy. *Proc. Natl. Acad. Sci. USA*. 119, e2114858118 (2022).
