# Supplementary material for: Transcriptional adaptation upregulates utrophin in Duchenne muscular dystrophy
Source: Nature. 2025 Feb 12;639(8054):493–502. doi: 10.1038/s41586-024-08539-x (PMC11903304; doi:10.1038/s41586-024-08539-x)
Supplement: Supplementary file 2 — Reporting Summary [file 41586_2024_8539_MOESM2_ESM.pdf]

nature portfolio

Corresponding author(s): Didier Y.R. Stainier

Last updated by author(s): Dec 4, 2024

Reporting Summary

Nature Portfolio wishes to improve the reproducibility of the work that we publish. This form provides structure for consistency and transparency in reporting. For further information on Nature Portfolio policies, see our Editorial Policies and the Editorial Policy Checklist.

Statistics

For all statistical analyses, confirm that the following items are present in the figure legend, table legend, main text, or Methods section.

n/a	Confirmed	
☐	☒	The exact sample size (*n*) for each experimental group/condition, given as a discrete number and unit of measurement
☐	☒	A statement on whether measurements were taken from distinct samples or whether the same sample was measured repeatedly
☐	☒	The statistical test(s) used AND whether they are one- or two-sided<br>*Only common tests should be described solely by name; describe more complex techniques in the Methods section.*
☐	☒	A description of all covariates tested
☐	☒	A description of any assumptions or corrections, such as tests of normality and adjustment for multiple comparisons
☐	☒	A full description of the statistical parameters including central tendency (e.g. means) or other basic estimates (e.g. regression coefficient) AND variation (e.g. standard deviation) or associated estimates of uncertainty (e.g. confidence intervals)
☐	☒	For null hypothesis testing, the test statistic (e.g. *F*, *t*, *r*) with confidence intervals, effect sizes, degrees of freedom and *P* value noted<br>*Give P values as exact values whenever suitable.*
☒	☐	For Bayesian analysis, information on the choice of priors and Markov chain Monte Carlo settings
☒	☐	For hierarchical and complex designs, identification of the appropriate level for tests and full reporting of outcomes
☒	☐	Estimates of effect sizes (e.g. Cohen's *d*, Pearson's *r*), indicating how they were calculated

Our web collection on statistics for biologists contains articles on many of the points above.

Software and code

Policy information about availability of computer code

Data collection	QuantStudio™ 7 Pro Real-Time PCR System, Image Lab Software (Bio-Rad)
Data analysis	GraphPad Prism 9, ImageJ

For manuscripts utilizing custom algorithms or software that are central to the research but not yet described in published literature, software must be made available to editors and reviewers. We strongly encourage code deposition in a community repository (e.g. GitHub). See the Nature Portfolio guidelines for submitting code & software for further information.

Data

Policy information about availability of data

All manuscripts must include a data availability statement. This statement should provide the following information, where applicable:

- Accession codes, unique identifiers, or web links for publicly available datasets
- A description of any restrictions on data availability
- For clinical datasets or third party data, please ensure that the statement adheres to our policy

The public dataset generated for this paper, PRJNA1187808, contains RNA-seq data from DMD PTC and WT HEK293T cells treated with TSA. Source data are provided with this paper.

Research involving human participants, their data, or biological material

Policy information about studies with human participants or human data. See also policy information about sex, gender (identity/presentation), and sexual orientation and race, ethnicity and racism.

Reporting on sex and gender	n/a
Reporting on race, ethnicity, or other socially relevant groupings	n/a
Population characteristics	n/a
Recruitment	n/a
Ethics oversight	n/a

Note that full information on the approval of the study protocol must also be provided in the manuscript.

Field-specific reporting

Please select the one below that is the best fit for your research. If you are not sure, read the appropriate sections before making your selection.

☒ Life sciences ☐ Behavioural & social sciences ☐ Ecological, evolutionary & environmental sciences

For a reference copy of the document with all sections, see nature.com/documents/nr-reporting-summary-flat.pdf

Life sciences study design

All studies must disclose on these points even when the disclosure is negative.

Sample size	No statistical methods were used to predetermine sample size. Sample size was determined according to the minimal number of independent biological replicates that significantly identified an effect. For most experiments, we analyzed at least 3 sets of biologically independent samples.
Data exclusions	No data were excluded.
Replication	Replication was performed at least twice for each experiment.<br>Experiments were independently replicated, with biological and technical replicates.
Randomization	No randomization was performed in these experiments due to the specific requirements of the experimental setup, including the predetermined genotypes of the cells and the treatments administered. These constraints necessitated a focused approach rather than random assignment.
Blinding	Blinding was not performed due to the same constraints that precluded randomization, specifically the necessity to account for the predetermined genotypes of the cells and the specific treatments applied during the experiments.

Reporting for specific materials, systems and methods

We require information from authors about some types of materials, experimental systems and methods used in many studies. Here, indicate whether each material, system or method listed is relevant to your study. If you are not sure if a list item applies to your research, read the appropriate section before selecting a response.

Materials & experimental systems

n/a	Involved in the study	
☐	☒	Antibodies
☐	☒	Eukaryotic cell lines
☒	☐	Palaeontology and archaeology
☒	☐	Animals and other organisms
☒	☐	Clinical data
☒	☐	Dual use research of concern
☒	☐	Plants

Methods

n/a	Involved in the study	
☒	☐	ChIP-seq
☒	☐	Flow cytometry
☒	☐	MRI-based neuroimaging

Antibodies

Antibodies used	The following antibodies were used: anti-Dystrophin (Proteintech, 68120-1-Ig), anti-Utrophin (Proteintech, 29133-1-AP), anti-H3K9ac (Proteintech, 29133-1-AP), and anti-tubulin (Sigma, T6557).
Validation	All antibodies were obtained from the indicated commercial vendors and have been validated for the application by the manufacturer.

Eukaryotic cell lines

Policy information about cell lines and Sex and Gender in Research

Cell line source(s)	HEK293T cells were purchased from DSMZ.<br>The human myoblasts were supplied by the Institut de Myologie in Paris, under the authorization code AC-2019-3502.<br>The biopsies used for generating the cell lines were supplied by MyoBank, the tissue bank associated with the Institut de Myologie in Paris and affiliated with EuroBioBank. MyoBank is authorized by the French Ministry of Higher Education, Research, and Innovation to distribute human samples for research purposes.<br>Mouse C2C12 myoblasts were obtained from ATCC (#CRL-1772).<br>HAP1 cells were purchased from Horizon Discovery.<br>HeLa cells were obtained from Enzo Life Sciences.
Authentication	None of the cell lines were authenticated by the authors.
Mycoplasma contamination	All cell lines tested negative for mycoplasma contamination.
Commonly misidentified lines<br>(See ICLAC register)	No commonly misidentified cell lines were used.

Plants

Seed stocks	n/a
Novel plant genotypes	n/a
Authentication	n/a
